# Nanostructured Protein Surfaces Inspired by Spider Silk

**DOI:** 10.1002/adma.202508959

**Published:** 2025-07-28

**Authors:** Martin Humenik, Thomas Scheibel

**Affiliations:** ^1^ Department of Biomaterials University of Bayreuth Prof.‐Rüdiger‐Bormann‐Str. 1 95447 Bayreuth Germany; ^2^ Bayreuth Center of Material Science and Engineering (BayMat) University of Bayreuth Universitätsstr. 30 95440 Bayreuth Germany; ^3^ Bavarian Polymer Institute (BPI) University of Bayreuth Universitätsstr. 30 95440 Bayreuth Germany; ^4^ Bayreuth Center of Colloids and Interfaces (BZKG) University of Bayreuth Universitätsstr. 30 95440 Bayreuth Germany; ^5^ Bayreuth Center for Molecular Biosciences (BZMB) University of Bayreuth Universitätsstr. 30 95440 Bayreuth Germany

**Keywords:** coatings, functional materials, hydrogels, particles, self‐assembly, spinning

## Abstract

Spider silk is renowned for its exceptional mechanical properties, surpassing those of many natural and synthetic materials. This review focuses on biotechnologically produced recombinant spidroin variants inspired by well‐understood major ampullate spider silk proteins (=spidroins), which are role models for understanding molecular composition, architecture, and the nanoscopic and mesoscopic structures formed through self‐assembly and phase separation of spider silk fibers. The use of recombinant spidroins is explored to fabricate functionalized nanostructured surfaces, and molecular engineering is highlighted to tailor the interfacial properties of various morphologies, including particles, capsules, electrospun nanofibers, films/coatings, macroscopic nanofibril‐based hydrogels, and nanohydrogel coatings. One focus is on functionalization of spidroins with peptide tags enabling a variety of affinity‐based targets from cellular markers to inorganic nanoparticles, and allowing for instance specific drug delivery, cell accommodation in hydrogels, or bioselective immobilization of cells on surfaces. Furthermore, applying nanostructured spidroin coatings in combination with photo‐ and soft‐lithography techniques is demonstrated, which can be used to produce micro‐ and nanostructured patterns exhibiting confined, spidroin‐defined targeting, affinity, or repulsion properties.

## Graphical abstract

Harnessing recombinant spider silk technology, bioengineered spidroin variants enable the creation of functionalized nanostructured coatings with tunable affinity for specific targets, supporting a broad range of applications ‐ from antifouling surfaces and targeted drug delivery to advanced cell therapies and precision bio‐patterning via lithography.

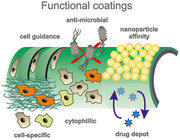

Spider silk attracts materials scientists primarily as a protein‐based fiber that outperforms most natural and synthetic materials in terms of mechanical performance. In recent years, the understanding of the relationship between structure and self‐assembly of spidroins, i.e., the protein constituting the fibers, has enabled their use in approaches implementing functional design and improving performance and functionality in bio‐inspired materials.^[^ ^1^, ^2^, ^3^, ^4^, ^5^, ^6^, ^7^ ^]^

This review explores recent advances in spidroin‐based and ‐inspired nanostructured material surfaces achieved by processing engineered spidroins through intrinsic self‐assembly and/or phase separation at interfaces. These nanostructured surfaces are significantly determining the performance of the resulting materials and devices.

## 1 Hierarchical Structures of Major Ampullate Spider Silk

**Figure 1:**
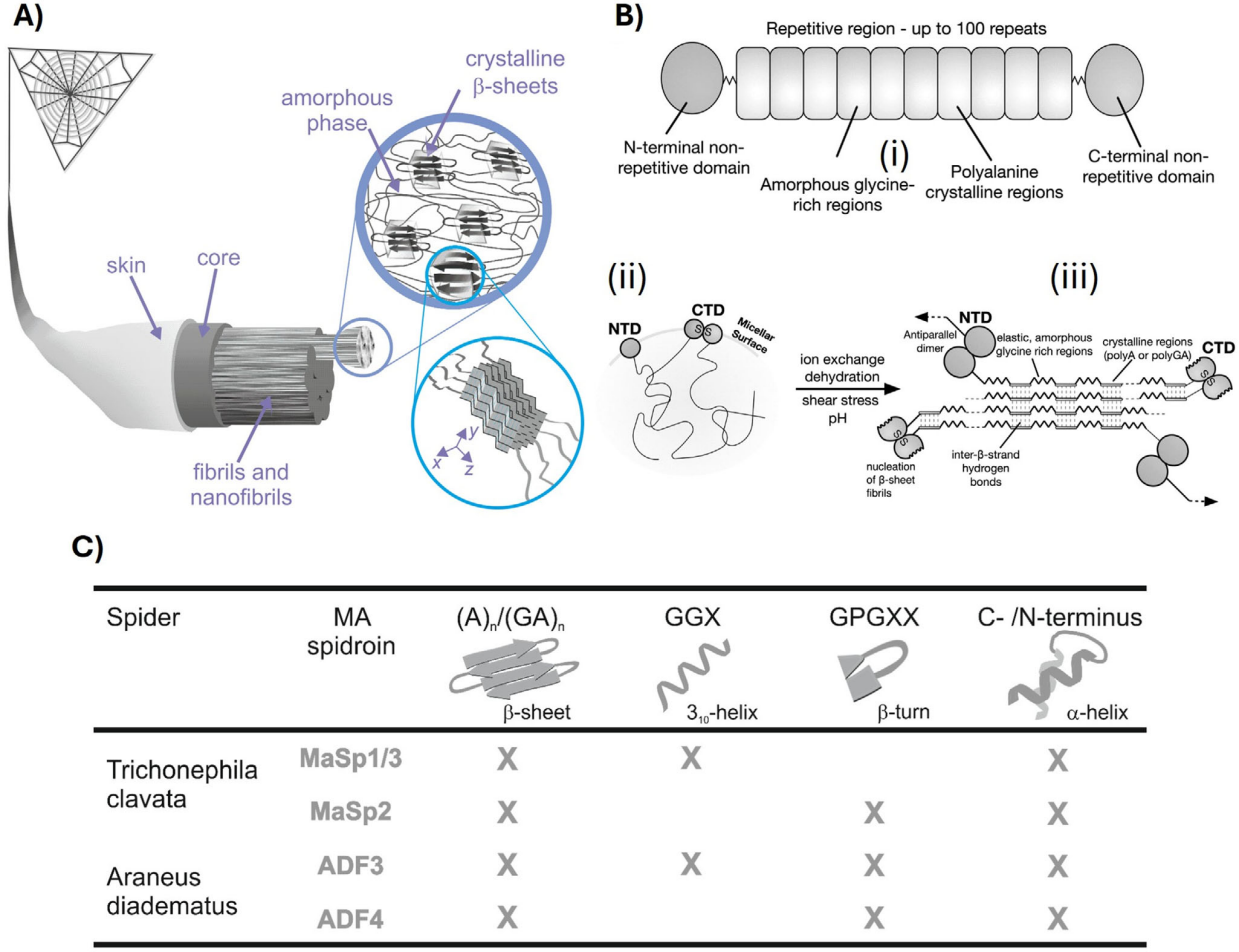
A) Hierarchical structure of MA silk. On the macroscale, a skin‐core structure is recognized, and on the sub‐microscale, 100 nm‐thick fibrils composed of entangled protofibrils with diameters of ≈10 nm are aligned with the overall fiber axis. At the nanoscale, β‐sheet crystallites are aligned with the fiber axis and embedded in an amorphous phase. The crystallites are composed of intramolecularly folded poly‐Ala stretches in an antiparallel β‐sheet configuration stabilized via H‐bonds. Further intermolecular stacking of the β‐sheets lead to densely packed crystalline units via hydrophobic off‐plain oriented Alanine residues. The unit size (x,y,z) and orientation has been determined using x‐ray diffraction. Reproduced with permission from Refs. [55, 272]. B) Schematic representation of MaSps. (i) Their sequences consist of a large repetitive core domain and flanking non‐repetitive N‐ and C‐terminal domains (NTDs and CTDs, respectively). (ii) Soluble spidroins are stored in higher order structures resembling micelles. NTDs are monomeric and CTDs form permanent parallel dimers stabilized by a disulfide bond at storage conditions. It is hypothesized that the highly soluble NTD‐ and CTD‐ moieties are located at the micelle surface, whereas the amphiphilic repetitive domains show prevalently random coil conformation facing inside. (iii) Shear forces upon elongational flow, acidification, and salt exchange in the spinning duct trigger assembly of the spidroins into β‐sheet rich aligned fibrils yielding an insoluble silk fiber at the end of the spinning duct. Reproduced with permission from Ref. [273]. C) Repetitive sequence motifs and intentional secondary structures as present in *Trichonephila clavata* and *Araneus diadematus* MA silk (the presence in the respective spidroin is indicated by “X”). Reproduced with permission from Ref. [272].

Major ampullate (MA) spider silk fibers outperform most natural and synthetic counterparts concerning their toughness.^[^ ^8^, ^9^ ^]^ This specific mechanical feature results from nano‐ and mesoscaled hierarchical structures within the fibers (**Figure** **1**A) obtained in a self‐assembly process during fiber spinning.^[^ ^8^, ^10^, ^11^, ^12^ ^]^

The MA silk is composed of proteins named major ampullate spidroins (MaSps, see the general setup in Figure 1B). The main subtypes, MaSp1‐3, are large proteins with molecular weights often exceeding 250 kDa.^[^ ^8^, ^10^ ^]^ Repetitive core domains (RCD) comprise polyalanine (An, n = 4–8) and distinct glycine‐rich motifs (Figure 1C) organized in units which can be repeated over 100 times within the RCD.^[^ ^13^, ^14^, ^15^ ^]^ The mixture of MaSps depends on the spider species, and environmental conditions can trigger spiders to adjust this mixture it in order to alter the tensile properties of the fibers.^[^ ^11^, ^15^, ^16^ ^]^ This adaptability could be reproduced *in vitr*o in experiments with biomimetic fibers made from heterogonous mixtures of recombinant MaSps,^[^ ^17^, ^18^ ^]^ highlighting that spiders use both, optimized sequences^[^ ^19^, ^20^ ^]^ and “polymer blending”, to achieve the appropriate mechanical properties of a fiber. However, modern genomic and proteomic analyses revealed numerous additional minor protein constituents and posttranslational modifications in MA silk,^[^ ^15^, ^21^, ^22^ ^]^ the roles of which remain to be clarified in fiber mechanics.

Spidroins are secreted as soluble proteins and stored at concentrations exceeding 50% w/v at neutral pH in so called spinning dopes.^[^ ^8^, ^11^ ^]^ Studies on soluble recombinant proteins comprising only RCD‐based sequences have shown predominantly intrinsically unstructured features.^[^ ^23^, ^24^, ^25^ ^]^ In contrast, terminal domains were shown to be folded structures consisting of five‐helix‐bundles.^[^ ^26^, ^27^, ^28^ ^]^ The predominantly intrinsically unstructured features of MaSps could also be shown in *ex vivo* nuclear magnetic resonance (NMR) studies^[^ ^29^, ^30^, ^31^ ^]^

**Figure 2:**
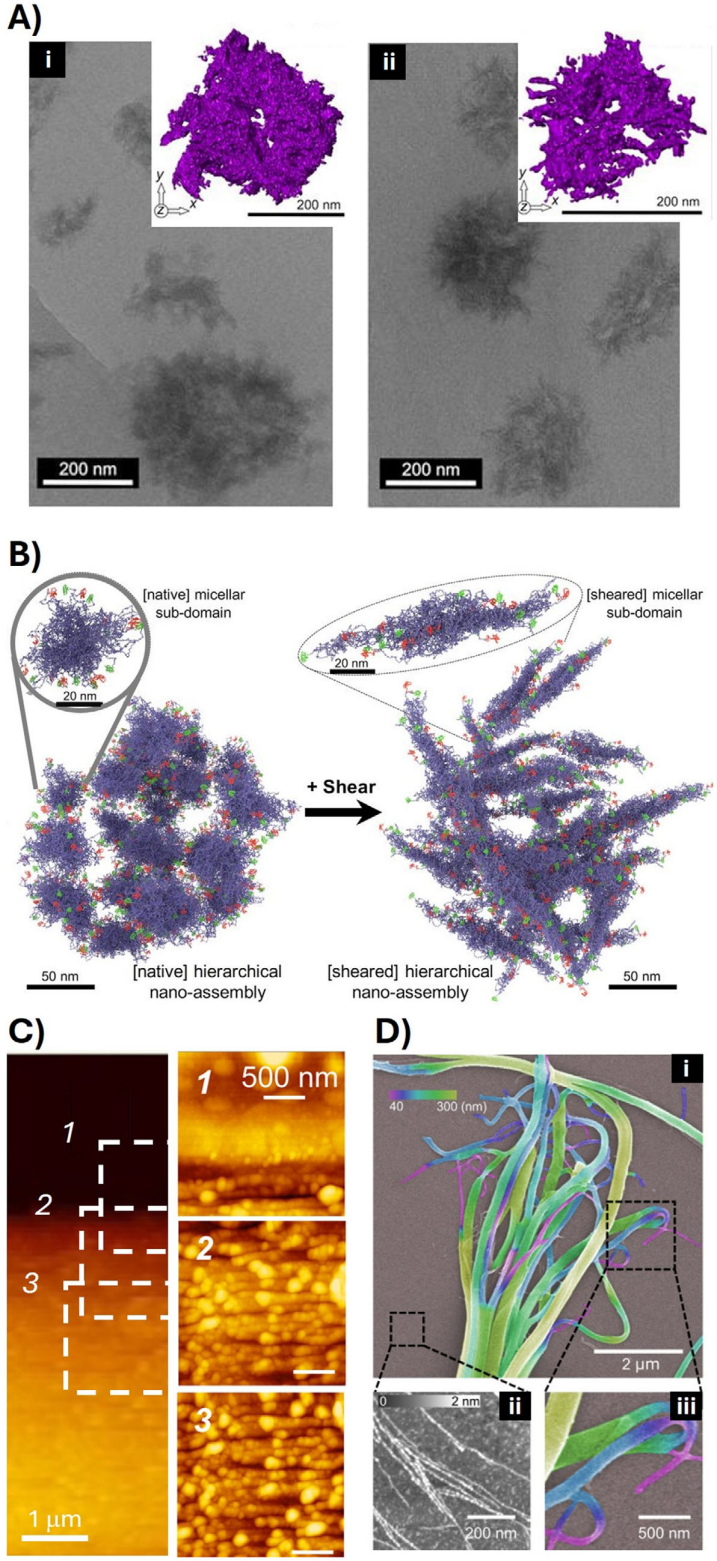
Supramolecular structure of MA silk. A‐(i) Dope of *Latrodectus hesperus* MA silk imaged using cryo‐TEM showed spherical (200–400 nm) micellar protein assemblies. Inserts highlight the 3D isosurface rendering of the assemblies. (ii) The spidroin assemblies imaged after shear showed spherical hierarchical assemblies composed of interwoven fibrillar subdomains. B) Graphical interpretation of cryo‐TEM and diffusion NMR data – in the micellar sub‐domain (gray circle), the NTDs and CTDs (green and red) form helical structures, whereas the long, repetitive domain (blue) is in a molten‐globule state (rH ≈ 20–25 nm) and is assembled into larger, spherical micellar structures (several hundred nanometers in diameter). When sheared, the initial transformation occurs into narrow, elongated fibrils that remain assembled (disordered and interwoven network) within the spherical hierarchical assemblies. A) and B) reproduced with permission from Ref. [32]. C) Longitudinal cross‐sectional topography of *T. clavata* MA silk imaged using AFM. The boxed regions on the fiber show the position of the enlargements 1–3. Box 1 further shows the border region between glue and silk fiber. The scans demonstrate the presence of granular microfibrils with a width of 113 ± 20 nm. Reproduced with permission from Ref. [38]. D) Disintegration of MA silk fiber after ultra‐sonication imaged using SEM (i and iii), and exfoliated nanofibrils are imaged using AFM of a representative area (ii). Reproduced with permission from Ref. [56].

Unter storage conditions, liquid crystals,^[^ ^8^ ^]^ nano‐ to submicroscopic micelles^[^ ^32^, ^33^ ^]^ and microscopic droplets due to liquid–liquid phase separation (LLPS)^[^ ^34^, ^35^, ^36^ ^]^ have been identified as well as combinations thereof.^[^ ^37^, ^38^ ^]^ A consolidated view of separate theories, how this supramolecular structures contribute to the assembly and spinning process, have been provided recently as a concerted, transitional concept also highlighting implications on the technical spinning of artificial spidroins.^[^ ^39^ ^]^ The transition of fluid precursors in the dopes to solid fibrous materials is triggered by changes from neutral to acidic pH, elongational flow, chaotropic‐to‐kosmotropic ion exchange and dehydration along the spinning duct.^[^ ^40^, ^41^ ^]^ Upon these transitions, conformational changes lead to supramolecular nanoscopic assemblies of the structured building blocks occur (**Figure** **2**A,B).^[^ ^42^, ^43^, ^44^, ^45^, ^46^ ^]^ In this process, the N‐ and C‐terminal domains (NTDs and CTDs) have been recognized as important structural switches, as the NTDs form antiparallel dimers upon acidification, and the CTDs partially unfold due to shear forces exposing hydrophobic surfaces.^[^ ^27^, ^28^, ^47^, ^48^, ^49^ ^]^ Hence, the conformational switches of both domains lead to new interconnections in the protein network (Figure 1A(iii)).

The pre‐assembled micellar or granular structures in the dope are necessary for the correct formation of fibrils, which form due to elongational flow and longitudinal deformation of the globular structures across the tapering spinning channel (Figure 2A,B).^[^ ^32^ ^]^ The estimated fibril dimensions vary between 10 and 100 nm in diameter depending on the analytical method used (Figure 2C).^[^ ^38^, ^50^, ^51^, ^52^, ^53^, ^54^, ^55^ ^]^ Multiple nanoscale fibrils are further packed in a parallel orientation with the fiber axes, and it is possible in some specific cases to disassemble the fiber again into fibrils using physical or chemical energy input since they are not chemically crosslinked like other protein fibers (Figure 2D). This exfoliated fibrils could be used as nanomaterial building blocks.^[^ ^56^, ^57^, ^58^, ^59^ ^]^

The development of recombinant MaSps enabled not only to mimic natural fibers^[^ ^3^, ^36^, ^39^ ^]^ but also to self‐assemble other morphologies, such as hydrogels, particles, capsules, nanofibers, and coatings, some of which will be reviewed in the following paragraphs.

## 2 Controllable Surfaces Made of Spidroin‐Based Materials

Deciphering natural MaSp genes, designing of artificial MaSp sequences using recombinant DNA technology, and the production of recombinant MaSps variants in different host organisms became a feasible route for large‐scale MaSp production, otherwise hampered by spider cannibalism impacting farming, opening the way for material sciences and applications.^[^ ^2^, ^5^, ^60^, ^61^ ^]^

**Figure 3:**
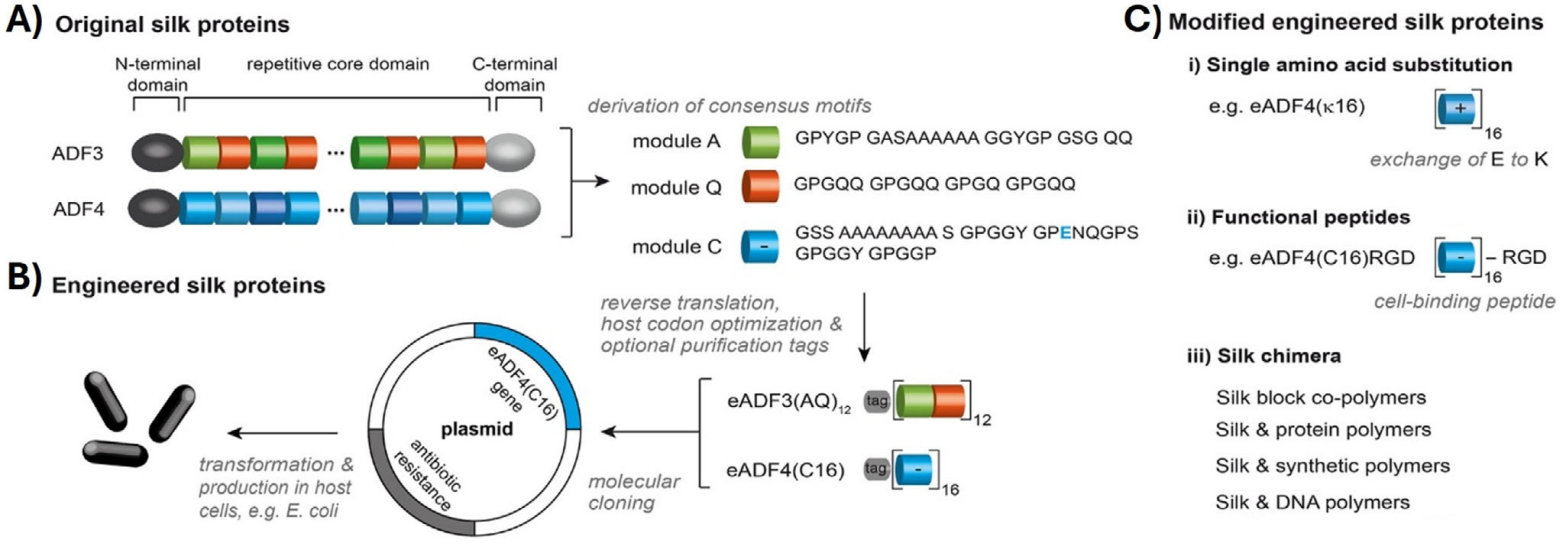
Schematic overview of genetic engineering of MaSps, based on the MaSp2 variants ADF3 and ADF4 of the European garden spider *A. diadematus*. A) Amino acid consensus motifs of the repetitive core regions were identified and back‐translated into synthetic DNA sequences, while adapting the codon usage to that of the host organism (e.g., Escherichia coli). B) Using a seamless cloning strategy, the DNA cassettes (modules) were multimerized into engineered silk genes. The sequences of the non‐repetitive C‐ and N‐ terminal regions could be amplified at this stage (e.g., using PCR), combined with the core domain, and then introduced in a host‐specific expression plasmid. C) A modular cloning system enables the generation of specific genetically engineered MaSps, e.g. (i) using point mutations to adjust the net charge of MaSp variants, and (ii) to add peptide tags or enzyme sequences for functionalization. (iii) Further, chemical conjugations of distinct amino acid residues enable extended functionalization with bio‐ and synthetic polymers. Hence, the modification toolbox allows tailoring the resulting spider silk‐based materials for various applications. Reproduced with permission from Refs. [274].

One important step toward biotechnological production of MaSps was genetic engineering including the design of consensus sequences of respective repeating units with DNA sequences adjusted to the codon usage of the production organism. Next, the consensus sequences were multimerized to the required extent and finally furnished on demand with further sequences, e.g. such of the terminal domains. The final gene was implemented into a vector DNA (plasmid) controlling the expression in the chosen host organism.^[^ ^62^ ^]^ The engineering of spidroins is exemplarily shown for MaSp2 variants of *Araneus diadematus* (**Figure** **3**A,B). Engineered MaSps have been commonly used in biomimetic spinning processes to obtain artificial spider silk fibers with tunable mechanical properties.^[^ ^3^, ^4^, ^5^, ^6^, ^63^ ^]^

However, recombinant MaSps can also be used in processing of non‐fibrous materials with nano‐ and micro‐structured surfaces employing the intrinsic properties of the proteins. The morphologies and distribution thereof vary upon used sequences as well as employed processing conditions including self‐assembly from aqueous buffers, processing from organic solvents or implementation of advanced photo‐ and soft‐lithography techniques. Further, the recombinant MaSps can be functionalized (Figure 3C) including genetic fusions with sequences of peptide tags to target for example cells, or chemical modifications to create hybrid molecules allowing surface addressability not achievable by natural amino acid residues.

### 2.1 Surface Control of Spider Silk Particles

**Figure 4:**
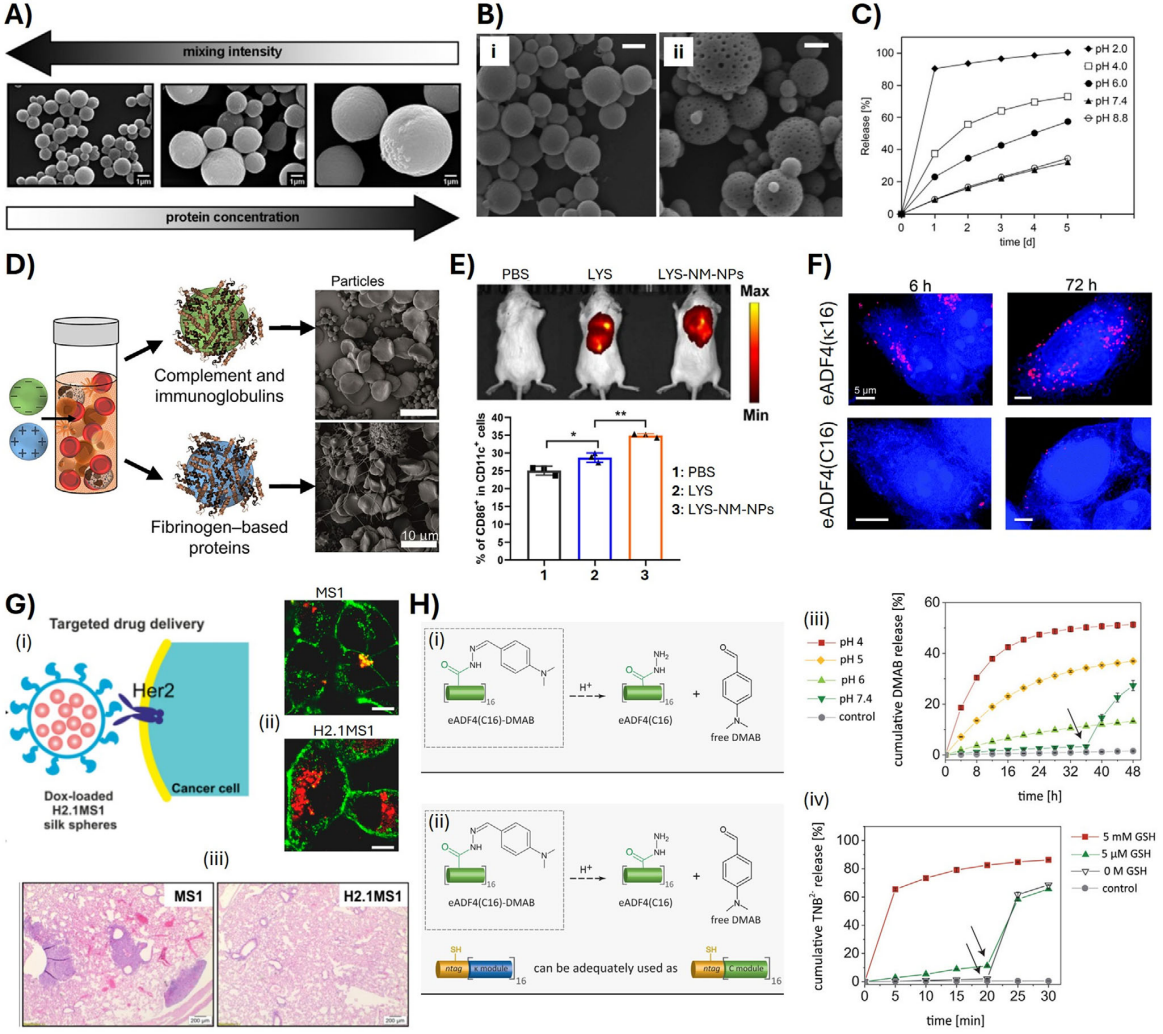
Properties of spider silk particles and their applications. A) and B) Size and morphology of the particles can be adjusted. A) Mixing intensity and spidroin concentration have an impact on the particle diameters. Reproduced with permission from Ref. [67]. B) Non‐porous (i) and porous (ii) morphologies can be achieved depending on the initial purification conditions. Reproduced with permission from Ref. [71]. C) Release profiles of ethacridine from negatively charged eADF4(C16)‐based particles as a function of pH. At pH values above 7, the positively charged drug strongly interacts with the negatively charged protein resulting in slow release, whereas decreasing the pH and protonation of the spidroin's Glu‐residues results in burst‐release at pH values below the protein's pI of 3.9. Reproduced with permission from Ref. [86]. D) Proteomic analyses revealed that different proteins from human blood can interact with spidroin particles, depending on the particle's surface charge. Whereas the negatively charged eADF4(C16) particles predominantly interact with complement and immunoglobulins resulting in hemocompatibility, the positively charged eADF4(κ16) particles bind mainly fibrinogen triggering blood coagulation. Reproduced with permission from Ref. [93]. E) In vivo immunization using particles produced from a negatively charged *A. ventricosus* MiSp variant loaded with lysozyme. The transport into draining lymph nodes and the induction of dendritic cell maturation was compared using PBS, free lysozyme (LYS) and loaded spidroin particles (LYS‐NM‐NPs). Upper panel: fluorescence images of Cy5.5‐labeled lysozyme at the injection site at time 0. Lower panel: the relative proportion of cells positive for CD86 surface markers was measured using flow cytometry showing the highest expression of CD86 in case of the LYS‐NM‐NPs‐treated group at 48 h after injection. Reproduced with permission from Ref. [73]. F) Fluorescence microscopy images of HeLa cells incubated in the presence of spidroin particles for 6 to 72 h, as indicated, showing significantly higher cellular uptake of the positively charged eADF4(κ16)‐particles in comparison to the negatively charged eADF4(C16) ones. Scale bars: 5 µm. Reproduced with permission from Ref. [75]. G‐(i) Schematic representation of particles made of the anti‐Her2 peptide‐tagged spidroin variant (H2.1MS1) delivering doxorubicin to Her2‐positive cancer cells. (ii) In vitro delivery of the H2.1MS1‐particles as well as non‐functionalized particles (MS1) to Her2 positive breast cancer cells, visualized using fluorescence microscopy. (iii) Comparison of the therapeutic effect of DOX delivered in the non‐functionalized and functionalized spidroin particles in a Her2(+) breast cancer metastasis mouse model after 20 days of treatment. Scale bar: 200 µm. Reproduced with permission from Ref. [98]. H) Model drug release. (i) For pH‐sensitive release, the carboxylic groups of eADF4(C16) were activated using 1‐ethyl‐3‐(3‐dimethylaminopropyl)carbodiimide (EDC) to bind hydrazine, which reacted with the aldehyde moiety of para‐dimethylaminobenzaldehyde (DMAB). At acidic conditions, the covalent hydrazide bond hydrolyzed, and the bound substance was released. (ii) For the redox‐sensitive system, ntag^Cys^ variants of spidroins containing a single cysteine residue were employed (negatively charged eADF4(C16) as well as positively charged eADF4(κ16) variants can be used). The reduced sulfhydryl groups of the ntag^Cys^ were treated with 5,5′‐dithiobis‐(2‐nitrobenzoic acid) (DTNB) to couple a 2‐nitro‐5‐thiobenzoate (TNB) moiety with the respective spidroins. At reducing conditions, the (TNB)^‐^ is released, followed by ionization yielding the anion (TNB)^2–^. (iii) Cumulative DMAB release from the hydrazide‐modified eADF4(C16) particles at various pH values. To confirm the ability to trigger the drug release, the pH of the release medium was changed from pH 7.4 to 4 after 36 h, as indicated by arrows. (iv) Cumulative TNB^2–^ release from ntag^Cys^‐eADF4(C16) particles with and w/o glutathione (GSH) (5 mM). To confirm the ability to trigger drug release, the release medium of all measurements was changed to physiological 5 µM GSH after 20 min, as indicated by arrows. Reproduced with permission from Ref. [101].

Concentration of phosphate ions above 400 mm can promote “salting‐out” of spidroins^[^ ^64^, ^65^ ^]^ and induce particle formation in aqueous buffers, as demonstrated for different recombinant spidroin variants, such as eADF4(C16) based on a consensus sequence originating from the repetitive domain of one MsSp2 derivative (ADF4) of the European garden spider *A. diadematus*,^[^ ^23^, ^66^, ^67^, ^68^ ^]^ *Trichonephila clavipes* variants MaSp1 (MS1)^[^ ^69^, ^70^ ^]^ and MaSp2 (MS2),^[^ ^71^ ^]^ *A. ventricosus* MaSp4 (M4R2),^[^ ^72^ ^]^ MiSp^[^ ^73^ ^]^ as well as for the NTD of aciniform silk (rAcSp2).^[^ ^74^ ^]^ Self‐assembly of the spidroins into round‐shaped particles is initiated by a liquid–liquid phase separation, transformation into β‐sheet rich structures and nucleus formation within the protein‐rich phase, followed by a fast growth of the particle at a millisecond time scale.^[^ ^68^ ^]^ All spidroin particles revealed high content of β‐sheets, which render them thermodynamically highly stable.^[^ ^66^ ^]^ Diameters of particles salted‐out from aqueous buffers ranged from 200 nm up to 10 µm and depended on the protein and phosphate concentration as well as the mixing intensity (**Figure** **4**A). The lower the protein concentration and the higher the Reynolds number, the smaller were the particle diameters.^[^ ^67^, ^71^ ^]^ The spherical morphology of the particles improved with increasing phosphate concentrations as well as with increased pH.^[^ ^67^, ^69^, ^71^ ^]^ To reach nanoparticle dimensions (< 100 nm in diameter), however, ionic liquids were required as a solvent.^[^ ^75^ ^]^ Non‐aqueous routes have been also used in case of the engineered tubuliform spidroin 1 (eTuSp1) from the black widow spider. A hexafluorisopropanol (HFIP)‐in‐oil method followed by ethanol treatment and evaporation was used to produce respective particles.^[^ ^76^, ^77^ ^]^ Interestingly, it has been shown that the purification method of the underlying spidroins, e.g. thermal or acidic extraction, may affect the particle size, morphology, zeta potential and drug loading capacity (Figure 4B).^[^ ^71^ ^]^ Further, an automated particle production process has been achieved under controllable and repeatable conditions using micromixing in combination with ultrafiltration.^[^ ^78^ ^]^

Spidroin particles substantially swell upon hydration (swelling factor of 2.3) inducing a drastic drop in elastic moduli from GPa in the dry state to MPa in the wet state^[^ ^79^ ^]^ dependent on the molecular weight of the used spidroins. Combination of force measurements using a colloidal probe technique and analysis of electrophoretic mobility revealed a polymer‐brush‐like surface, i.e., an ion‐permeable particle interface protruding several tenths of nm into the solution.^[^ ^80^, ^81^ ^]^ This polymer‐brush‐like chains represent an active surface which can nucleate further protein assembly for example in form of fibrils growing from the particle surface.^[^ ^82^, ^83^ ^]^

#### 2.1.1 Tunning of Spidroin Particle Properties Using Sequence Design

##### Charge Adjustment of Spidroins for Explicit Drug Delivery

The surface net charge of a particle plays a crucial role in drug loading as well as in drug release. Therefore, the sequence net charge of spidroins enables the adjustment of applications. For example, using the positively charged *T. clavipes* MaSp1 and negatively charged MaSp2^[^ ^84^ ^]^ or the positively charged eADF4(κ16) and negatively charged eADF4(C16)^[^ ^75^ ^]^ determined their suitability for various drug delivery applications, concerning the possibility of selecting drugs to be formulated.^[^ ^85^, ^86^, ^87^ ^]^

The amphiphilic properties of eADF4(C16) could be further used to stabilize non‐charged highly hydrophobic substances such as carotenoids.^[^ ^88^ ^]^ More commonly, the protein polyanionic properties were used to effectively load positively charged small molecular weight drugs by diffusion, whereas constant release rates at physiological conditions and accelerated rates in acidic environments were realized in vitro (Figure 4C).^[^ ^66^, ^86^ ^]^ The negative charge of the eADF4(C16) has also been used to control loading and release of high molecular weight cationic polyethylene imines^[^ ^89^ ^]^or biologicals. Bovine serum albumin (BSA), horse radish peroxidase (HRP), and lysozyme (LYS),^[^ ^90^, ^91^ ^]^ were not only absorbed on the particles’ surface, but also distributed within the eADF4(C16)‐particle matrix.^[^ ^91^ ^]^

The incorporation of LYS into a MiSp‐based delivery system has also been explored. The LYS‐loaded particles enhanced antigen‐specific immune responses in mouse models after subcutaneous or intramuscular administrations (Figure 4E) through a mechanism involving antigen‐depot effects at the injection site, long‐term antigen persistence, and efficient uptake by dendritic cells as well as internalization by lymph nodes.^[^ ^73^ ^]^

The antitumor peptide ChMAP‐28, which exhibits a positive net charge, was loaded on negatively charged M4R2‐ and rAcSp2‐based particles, showing high loading capacity and sustained‐release over 30 days.^[^ ^72^, ^74^ ^]^ The polycationic eADF4(κ16)‐based particles were loaded with negatively charged low‐molecular‐weight substances but also single stranded or anticancer doxorubicin (DOX)‐loaded double stranded nucleic acids.^[^ ^87^, ^89^ ^]^ Genetical manipulation allowed enhancing of drug loading and release profiles of several spidroins. Glu residues (E) were, for instance, introduced in the repetitive module of MS2 to lower the isoelectric point of the EMS2 variant (pI  =  3.15 vs 5.27), which impacted the loading and release efficiency of DOX in a pH depended manner.^[^ ^92^ ^]^ In case of eTuSp1, histidine residues were introduced into the silk sequence to increase the protein's pI to 4.8, hence allowing the controlled release of DOX at lysosomal acidic conditions.^[^ ^76^, ^77^ ^]^

Importantly, the surface charge of the particles can also enhance their cellular uptake. In this context, the polyanionic protein eADF4(C16) was functionalized with various positively charged cell penetrating peptide tags, and the cellular uptake of the respective particles was analyzed (Figure 4F).^[^ ^75^, ^89^ ^]^ The highest uptake was observed in the presence of a poly‐arginine tag (zeta potential −17.1 mV) and for the polycationic eADF4(κ16) (without a tag, zeta potential 13.2 mV). The uptake mostly occurred through clathrin‐mediated endocytosis for all used spider silk particles.^[^ ^75^ ^]^

Like most polymeric carrier systems, spidroin particles adsorb plasma proteins when in contact with blood, resulting in the formation of a biomolecular corona. The effect of surface net charge of particles made of recombinant spidroin variants on the biomolecular corona composition has been assessed using in‐depth proteomic analysis (Figure 4D).^[^ ^93^ ^]^ The positively charged spidroin eADF4(κ16) interacted predominantly with fibrinogen‐based proteins which correlated with the enhanced blood clotting activity. In contrast, negative surface charges of the unmodified eADF4(C16) and tagged eADF4(C16)E8G, eADF4(C16)RGD as well as eADF4(C16)R8G particles absorbed predominantly lactotransferrin and complement C3 resulting in blood clotting prevention. The peptide tags exposed on a particle's surface had only a minor influence, and the type of the corona was directed by the overall particle surface charge.^[^ ^93^ ^]^

##### Spidroin Fusion with Peptide Tags for Cell‐Specific Interactions

Fusion of recombinant spidroins with peptide tags can be done on a genetic basis to provide cell targeting function toward specific cellular receptors. For instance, the sequence of the integrin‐binding peptide RGD has been fused with eADF4(C16) to enhance uptake of respective particles made thereof in mammalian cells.^[^ ^75^, ^89^, ^94^ ^]^ Upon addition of a short peptide antigen (amino acid sequence: SIINFEKL), the modified ADF4(C16) particles were also utilized to target dendritic cells, leading to successful activation of cytotoxic T‐cells, whereas no signs of immunotoxicity or unspecific immunostimulatory activity were observed.^[^ ^95^ ^]^ In other studies, human epidermal growth factor receptor 2 (Her2) binding peptides (H2.1 or H2.2) were fused with MS1 and MS2 variants (Figure 4G). Internalization in Her2‐positive cancer cells showed the entry through clathrin‐ and caveola‐dependent endocytosis pathways in vitro and in vivo.^[^ ^84^, ^85^, ^96^, ^97^, ^98^ ^]^ Further tuning of the Her2‐target system improved the efficacy of drug loading into the particles upon incorporating a DOX‐affinity peptide into the spidroins.^[^ ^99^ ^]^ Moreover, the incorporation of cell matrix metalloprotease (MMP)‐responsive recognition sequences between the RCD‐modules of the MS1 and MS2‐variants enabled higher levels of degradation and, hence, release of DOX in the presence of cancer cells.^[^ ^100^ ^]^

#### 2.1.2 Chemical Modification of Particles for Controlled Drug Release

Chemical strategies have been developed to actively bind and release anticancer drugs upon chemical triggers using spider silk particles (Figure 4H). Through genetic engineering, the eADF4‐based spidroin sequences were extended at the amino‐terminus with a short amino acid tag comprising a single cysteine (ntag^Cys^), hence being the only cysteine residue in the sequence. The anticancer drug 6‐mercaptopurine was oxidatively coupled to ntag^Cys^‐eADF4(κ16) particles, and the release was demonstrated reducing the redox‐sensitive disulfide bond in the presence of glutathione (GSH) at concentrations mimicking the intracellular redox environment. In a second approach, the ketone group in DOX was coupled to chemically modified hydrazine‐functionalized Glu‐residues of eADF4(C16). Upon lowering the pH, a slow, sustained drug release was possible.^[^ ^101^ ^]^

Utilization of genetically and chemically functionalized spidroins in formulations and applications of particles is summarized in **Table** **1**.

**Table 1:** Recombinant spidroins used in processing nanostructured surfaces in electrospun nanofibers, films and self‐assembled nanofibrillar coatings.

*Origin*/recombinant silk protein	Functionalization	Morphology	Gained function/proposed application	Refs.
*A. ventricosus* MiSp / (NM (RCD+NTD)	negatively charged	particles	lysozyme loading and immunization in vivo	[73]
MaSp4/(M4Rx)			delivery of antibacterial/anticancer peptide ChMAP‐28 (GRFKRFRKKLKRLWHKVGPFVGPILHY)	[72]
*L. hesperus* Tubuliform spidroin1/eTuSp1	negatively charged (His)_6_‐tag		in vitro loading and release of Dox at lysosomal pH	[76, 77]
*A. diadematus* MaSp2 / ADF4(C16)	unmodified C‐module, (C16) polyanion, single charged Glu‐residue in each module		loading and release of positively charged drugs	[66, 86]
			amphiphilic – formulation of hydrophobic drugs	[88]
			preventing blood clotting	[93]
			loading and release of biologicals	[90, 91]
		electrospun nanofibers and meshes	filters	[116, 117, 118, 123]
			yarns	[109, 110]
			loading of biologicals	[115]
			enhanced cell adhesion and vascularization – tissue engineering	[108, 122]
			neuronal conduits	[121]
		coatings	low mammalian cell adhesion, decreased immune response, decreased scaffold encapsulation	[153]
			microbe repulsive properties, inhibit infestation of biofilms	[183]
			Inhibit blood coagulation/increased hemocompatibility of implants	[93, 155]
		self‐assembled nanofibrillar coatings	coatings of diverse materials/microbe repulsive properties	[227]
			nucleated assembly on spider silk layers/mammalian and cancer cell repulsive properties	[233, 265]
	Peptide tags: antigen peptide (SIINFEKL)	particles	immune response activation of dendritic cells	[95]
	R8G (RRRRRRRRG) Tat (RKKRRQRRR)		enhanced cellular uptake	[75]
	fibronectin derived RGD (GGSGGRGDSPG)		integrin targeting enhanced cellular uptake	
		coatings	Integrin targeting, improved biocompatibility through increased cell adhesion and viability	[125, 193]
	(and unmodified)	hydrogels	biofabrication – 3D printed scaffolds with mammalian cells	[216, 219, 222]
			scaffold for vascularization	[223, 224]
	collagen derived KGD	coatings	cell‐specific attachment and growth of myoblasts – muscle tissue engineering	[125]
	laminin‐derived IKVAV and YIGSR	improved adhesion of Balb 3T3 fibroblasts, HUVEC as well as selected neuronal cells (e.g., NG108 hybrid and RN22 Schwann cells)		
	angiopoietin‐derived QHREDGS		improved adhesion of HaCaT keratinocytes, RN22 Schwann cells, NG108 hybrid cells, HUVECs, Balb 3T3, and BJ fibroblasts	
	fibronectin derived REDV		Improved endothelial cell selectivity – blood vessel tissue engineering	[194]
	osteo‐tag (GLRSKSKKFRRPDIQYPDATDEDITSHM) collagen binding domain from human osteopontin sialoN‐tag (DSSEENGNGDSSEEEEEEEETS) and sialoC‐tag (EDESDEEEEEEEEEE) present N‐ and C‐terminally, respectively, in bone sialoprotein		enhanced calcium phosphate formation, increased viability of MC3T3‐E1 mouse preosteoblasts/bone tissue engineering	[200]
	Chemically modified: eADF4(C16) ‐EDC/NHS based chemical conjugation with Glu residues		functionalization with azido linkers, click‐based immobilization of glycosides	[266]
			immobilization of enzymes and dyes	[143]
	*ntag^Cys^* (GCGGSGGGGSGGGG) *ntag^Cys^* ‐eADF4(C16)		unique site‐specific Cys residue, immobilization of maleimide modified AuNP	[146]
			immobilization of maleimide modified cyclo‐RGD	[125]
		particles	redox‐dependent coupling and release of drugs	[101]
	hydrazide (chemically modified Glu‐residue)	particles	pH‐dependent coupling and release of ketone and aldehyde modified drugs	
	azido linker (chemically modified N‐terminus)	self‐assembled nanofibrillar scaffolds	DBCO‐modified ssDNA strands and hybridization‐based immobilization of DNA‐modified cells	[233]
			DBCO‐modified anti‐cancer marker aptamers and cell‐specific immobilization of cancer cells	[265]
			DBCO modified anti‐thrombin aptamers – protective enzyme depots	[226]
ADF4(κ16)	unmodified: kappa module, (κ16) polycation, single aa substitution (Glu to Lys) in each module	particles	enhanced cellular uptake	^[^ ^75^ ^]^
			loading of negatively charged drugs and nucleic acids	[87, 89]
		coatings	good adhesion of cardiomyocytes	[188]
			enhanced blood coagulation/wound dressings	[155]
			good adhesion of cardiomyocytes	[188]
			enhanced blood coagulation/wound dressings	[155]
	peptide tags TiO_2_ binding peptide (QPYLFATDSLIK) Au binding peptide (WQVQVEVQVEVQVQVQV)		water splitting – hydrogen production upon light irradiation	[202]
	ntag^Cys^_			
		e‐spun meshes	Janus fibers with eADF4(C16), face selective binding of AuNP	[141]
*A. diadematus MaSp2*/eADF4(AQ)x	eADF4(AQ)12 and eADF4(AQ)24	coatings	microbe repulsive properties, inhibition of biofilm formation	[183]
*E. australis* MaSp1 RCD (Rep), *A. ventricosus* MiSp CTD/4Rep‐CT /	Peptide tags: fibronectin derived CTGRGDSPAC tag (FN4RepC)	particles	integrin targeting enhanced cellular uptake	[94]
		coatings	integrin targeting enhanced cellular adhesion and proliferation	[126]
		self‐assembled nanofibrillar scaffolds	enhanced viability of human primary cells	[228]
	IKVAV‐peptide	coatings	supported of Schwann cell adhesion and proliferation	[192]
	antimicrobial peptide magainin	self‐assembled nanofibrillar scaffolds	antimicrobial properties	[228]
	lysine tag (pLys) human galectin‐3 carbohydrate recognition domain (hGal3)		mucoadhesive properties	[235]
	affinity binding domains: ‐ Antibody‐binding Z domain ‐ Albumin‐binding domain Biotin‐binding domain M4		affinity‐based purification and sensing systems	[237, 238, 267]
	enzymes: sortase‐A tag (G)_5_		enzyme conjugation with DspB, PlySs2 and SAL‐1/Antimicrobial properties	[236, 239]
	xylanase		degrading the polysaccharide xylan	[268]
*E. australis* MaSp1 (NTD and RCD (Rep), *A. ventricosus* MiSp CTD/NT‐4Rep‐CT	vitronectin peptide (PQVTRGDVFTLP)	coatings	growth and differentiation of neuron model cell lines	[127]
	exchange of poly‐Ala with amyloid sequences (NT‐Amy‐6rep‐CT)	electrospun nanofibers and meshes	triboelectric power generation	[128]
*T. clavipes* MaSp1/6‐ and 15‐mer	unmodified and modified with CRGD‐peptide	electrospun nanofibers and meshes	growth and differentiation of neuron model cell lines	[124]
	modified with peptide tags: antimicrobial peptide human neutrophil defensins (HNP) 1, 2, 4, and Hepcidin	coatings	antimicrobial activity	[150, 196, 197]
	Ag affinity peptides (NPSSLFRYLPSD, WSWRSPTPHVVT, KSLSRHDHIHHH)			[201]
	silica‐binding peptide R5 (SSKKSGSYSGSKGSKRRIL) R5 multimer (silaffin)		silica biomineralization, bone tissue engineering	[203, 204]
	hydroxyapatite (HA) binding domain VTKHLNQISQSY (VTK)		HA biomineralization, bone tissue engineering	[205]
	carboxyl terminal domain of dentin matrix protein 1 (CDMP1)			[269]
	bone sialoprotein (BSP)			[199]
*T. clavipes* MaSp1 and MaSp2/MS1, MS2	unmodified MS2 – negatively charged MS1 – positively charged	particles	blended particles, delivery of chemotherapeutics mitoxantrone and etoposide	[85]
	Peptide tags: Her2‐binding peptides, H2.1 (MYWGDSHWLQYWYET H2.2 (LTVSPWY)		targeting Her2 positive cancer cells in vitro and in vivo DOX delivery	[84, 96, 97, 98]
	DOX‐binding peptide in DOXMS2 (LWSPWYGGSW)		enhanced DOX binding	[99]
	metalloprotease cleavage sites: MMP2.2 (ESLAYYTARSLSRLTATS) MMP2.9 (RSLSRLTAKGPRQITATS)		enhanced biodegradation and release of DOX	[100]
*A. trifasciata* AcSp1 RCD (W) and MaSp2 CDT/W_2_C_ma2_	NGF‐binding motifs (NERALTL)	coatings	sequestration of nerve growth factor‐β (NGF), enhancing differentiation and neurite outgrowth in PC12 rat neuronal cells	[195]
*T. clavata* MaSp1/cp19k‐MaSp1	barnacle cement protein cp19k	Electrospun nanofibers and meshes	adhesive function	[138]
*Origin unknown /* GMCDRSSP‐IgF‐1	C‐terminal insulin‐like growth factor 1 (IGF‐1), intramodular *TEV* cleavage site + thrombin cleavage site (*ENLYFQG* LVPRGS)		enzymatically controlled cleavage and release of IGF and enhanced viability of endothelial progenitor cells (EPCs)	[270]

### 2.2 Electrospinning of Spidroins to achieve Nanostructured Surfaces

Electrospinning (ES) is a versatile fabrication technique utilizing electrostatic forces to produce fibers from polymer solutions or melts with dimensions ranging from the micrometer down to the nanometer scale. The high surface‐to‐volume ratio of the achieved fiber meshes make them highly interesting for various medical and technical applications.

**Figure 5:**
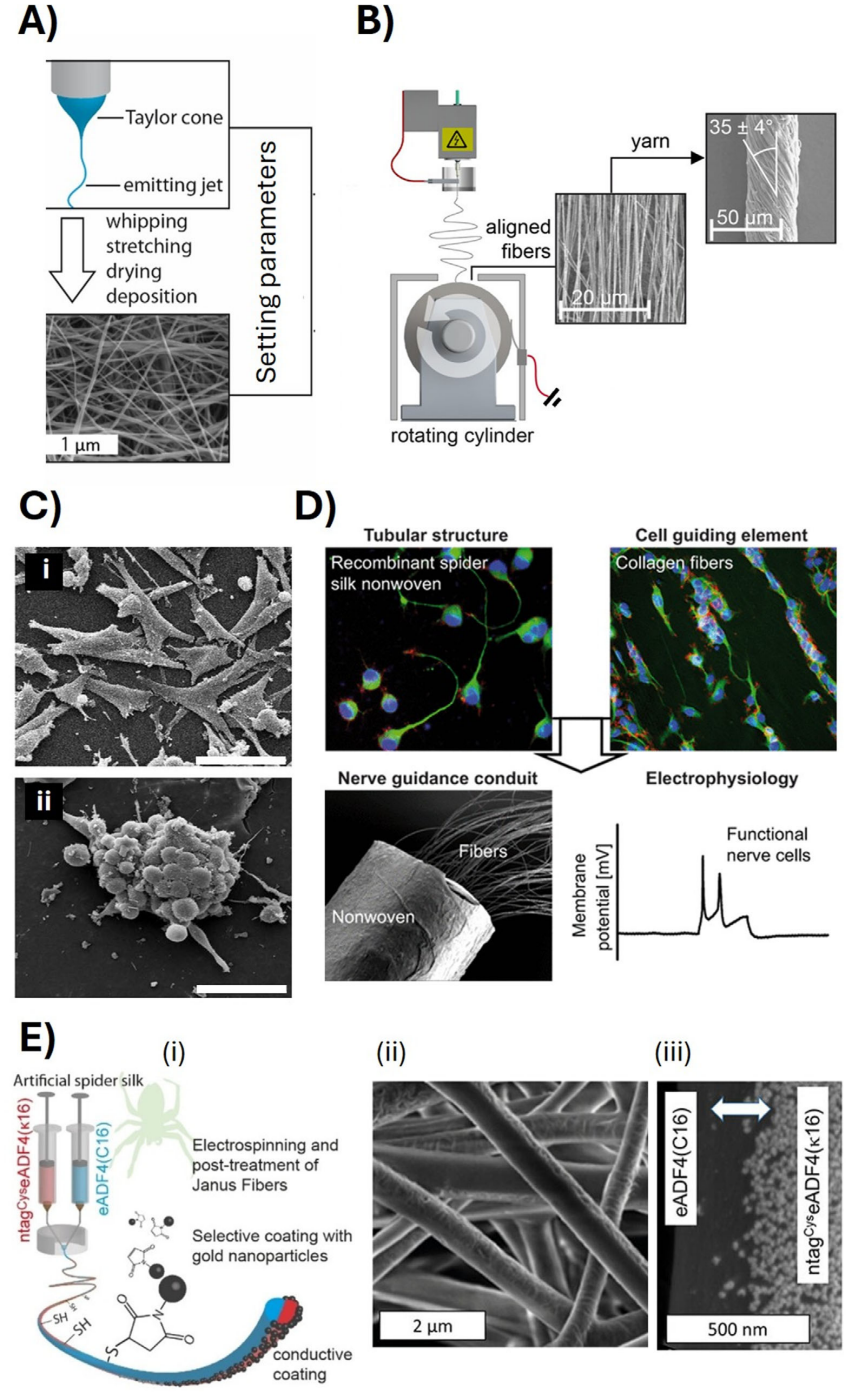
A) Schematic of a needle tip with the forming Taylor cone and a fiber jet as seen in a classical electrospinning setup. Adjusting diverse conditions (protein/polymer concentration, voltage, distance to collector, flow rate, temperature, humidity) allow for control of the diameter of the randomly deposited fibers yielding a mesh structure. Reproduced with permission from Ref. [275]. B) Collecting the fibers on an rotating drum lead to an aligned morphology, which can be further transferred into yarns upon twisting. Reproduced with permissions from Ref. [109]. C) Morphology of spreading cells incubated on an electrospun mesh made of eADF4(C16). In contrast, the cells could not adhere on flat films made thereof. Reproduced with permission from Ref. [108]. D) An electrospun eADF4(C16) mesh‐based tube filled with microfluidically spun collagen fibers together with neuronal cells yielded a nerve guiding conduit resulting in nerve signal transmission. Reproduced with permission from Ref. [121]. E(i) ntag^Cys^eADF4(κ16) and eADF4(C16) spidroins were processed using a coaxial electrospinning setup yielding a two‐phases Janus fiber, enabling chemically selective coupling of maleimide‐modified gold nanoparticles on the one half of the fiber, as shown in the SEM micrograph (ii and iii). Reproduced with permission from Ref. [141].

Technically, a protein/polymer solution is extruded through a needle connected to a high voltage supply (0–30 kV).^[^ ^102^, ^103^, ^104^, ^105^ ^]^ At the droplet's interphase, electrostatic repulsion forms a Taylor cone due to the strong electric field, which results in a stable jet, if electrostatic forces overcome the surface tension and the solution's molecular cohesion and chain entanglement are high enough.^[^ ^106^ ^]^ The charged jet undergoes whipping instabilities in the electric field, further stretching and solidifying the fiber due to fast solvent evaporation.^[^ ^107^ ^]^ The dried fibers are deposited on an oppositely charged collector, e.g. in a random order^[^ ^108^ ^]^ as nonwoven meshes, in aligned morphology, e.g. on a rotating drum,^[^ ^109^ ^]^ or in yarns^[^ ^110^ ^]^ (**Figure** **5**A,B). The fiber diameters are determined by the properties of the spinning dope (protein/polymer concentration, viscosity, conductivity, volatility, and surface tension of the solvent) and the process parameters (voltage, distance to collector, flow rate, temperature, humidity).^[^ ^104^, ^105^ ^]^

Native spider silk fibers have been collected, solubilized in organic solvents, and the spinning dopes were subjected to electrospinning to compare the material properties of the electrospun fibers with that of the natural ones concerning β‐sheet structure content, mechanical features and/or biological properties.^[^ ^111^, ^112^, ^113^ ^]^ One of the best studied recombinant spidroins in term of electrospinning is eADF4(C16). Adjusting the spinning dope concentration enabled a broad range of fiber diameters (0.1–1.0 µm).^[^ ^108^ ^]^ HFIP, hexafluoroacetone or formic acid‐based spinning dopes have been used, although, the latter is highly acidic which could degrade the peptide backbone or cause chemical changes, such as formylation upon prolonged incubation times, thus impacting the reproducibility of the spinning process and as well as the final fiber properties.^[^ ^114^ ^]^ Utilization of aqueous buffers for the spinning dope formulation can avoid these issues and even allows for the incorporation of biologically active and sensitive components in the spinning process.^[^ ^115^ ^]^ Importantly, as‐spun spidroin fibers are water soluble due to a low β‐sheet and a high α‐helical and random coil content. To stabilize the fiber meshes and, at the same time, to keep their morphology, the increase in β‐sheet content could be triggered using post‐treatment (PT) procedures involving vapor of alcohols or increased temperature as well as a combination thereof.^[^ ^108^, ^109^, ^111^, ^115^, ^116^ ^]^ Nano‐mechanical measurements based on lateral force microscopy showed that the post‐treatment yielding increased crystallinity, and hydration remarkably increased the fibers’ toughness up to a level of the natural fiber (152 ± 36 MJ m^−3^).^[^ ^109^ ^]^

In an advanced setup, centrifugal spinning was combined with electrospinning (CES), which can yield a high‐throughput large surface coverage with nanofiber‐based meshes due to production rates 1000 times higher in comparison to classical ES.^[^ ^117^, ^118^ ^]^ Further, the fiber diameters produced by CES were smaller than that by ES at comparable spinning dope conditions with advantages in, e.g. air‐filtration applications.^[^ ^104^ ^]^

In terms of biomedical applications, it has been shown that the adhesion and proliferation of mammalian cells on electrospun nanostructured meshes made of eADF4(C16) is significantly improved in comparison to that on flat coatings (Figure 5C). Interestingly, increasing the fiber diameter from 150 nm to 680 nm resulted in significant improvement of cell adhesion and proliferation, revealing a powerful tool to morphologically trigger cell growth on such surfaces.^[^ ^108^ ^]^ In vitro degradation experiments revealed that eADF4‐based nonwoven meshes exhibited a higher resistance toward proteolytic degradation in comparison to particles and films made of the same protein in tests using digestive enzymes as well as wound‐like enzymes.^[^ ^119^ ^]^

Nanoporous meshes have already been tested, e.g. as biodegradable wound dressings or in neuronal conduits.^[^ ^120^ ^]^ Concerning the latter, nonwoven meshes made of eADF4(C16) were processed into tubes incorporating aligned collagen fibers (Figure 5D). Neuronal differentiation was observed in a combined nerve guidance conduit. Differentiated NG108‐15 cells supported formation of neuronal networks and synapses, highlighting the combination of spidroin‐ and collagen‐based materials for peripheral nerve repair.^[^ ^121^ ^]^ In the latest study, vascularization and *de novo* tissue formation of fibrous matrices made from electro‐spun (ES) or wet‐spun (WS) eADF4(C16) have been analyzed using the rat arteriovenous (AV) loop model. The ES and the WS matrices showed both good biocompatibility and initiated tissue and vessel formation. The thinner electrospun fibers displayed a faster biodegradation and vascularization, indicating that the adjustment of the fiber diameter, i.e., scaffold porosity, is a valuable tool to optimize vascularization.^[^ ^122^ ^]^

eADF4(C16) fiber meshes have also been intensively investigated for technical applications, such as fine dust air filtration approaches, and the spider silk dust filter setups were more efficient in comparison to commercially available ones.^[^ ^116^, ^118^, ^123^ ^]^

#### 2.2.1 Enhancing Properties of Electrospun Spidroin Meshes

##### Functionalization of fibers using genetic engineering of spidroin sequences

Electrospun meshes made of RGD‐modified MaSp1 sequence, derived from *T. clavipes*, have been tested concerning bone tissue growth using human bone marrow‐derived mesenchymal stem cells (hMSCs). Surprisingly, nonwoven meshes of the spidroin without the RGD‐sequence showed higher bone‐related outcomes than with the RGD‐modified ones.^[^ ^124^ ^]^ Integration of the peptide motif directly into one of the RCD units likely resulted in insufficient exposure of the RGD group due to RCD folding within the fibers. This observation highlights the importance of careful sequence design, such as positioning the tags at the termini of the RCD and incorporating sufficiently long spacers, as demonstrated with other spidroins, which did not hinder the peptide tag functions.^[^ ^125^, ^126^, ^127^ ^]^ (see also Chapter 2.3.3.). In a different approach, applying genetic engineering to replace the poly‐Ala region with an amylogenic sequence, a low‐molecular‐weight biomimetic spidroin (Amy‐6rep) was developed showing excellent spinning performance and good tribopower generation capacity of the gained meshes.^[^ ^128^ ^]^ Establishment of aqueous electrospinning system of eADF4(C16) allowed the incorporation of active enzymes into the spinning dopes. One step ahead, the genetic fusion of an esterase enzyme with eADF4(C16) enabled efficient embedding of the bioactive component within the fibers.^[^ ^115^ ^]^

##### Blending of Spidroins with Synthetic Polymers

In regenerative medicine and tissue engineering, polymers like polyvinyl alcohol (PVA), polylactic acid (PLA) derivatives, and polycaprolactone (PCL) are commonly used as they are highly biocompatible and biodegradable. Upon blending these synthetic polymers with spidroins, the properties of electrospun scaffolds, such as mechanical features and biodegradability, could be adjusted for explicit applications, e.g. in wound dressings, for neural tissue regeneration or as vascular tissue scaffolds.^[^ ^129^, ^130^, ^131^, ^132^, ^133^, ^134^, ^135^ ^]^ Hence, using eADF4(C16) as a reinforcing agent in polylactide–polyglycolide (PLGA) blends, composite membranes were developed with improved spinnability, physicochemical, mechanical, and thermal properties. The spidroin reinforced meshes were noncytotoxic, showed no inflammatory response, enhanced cell migration and wound closure, making them ideal for advanced wound healing applications.^[^ ^136^ ^]^ Nonwoven meshes made of blends of recombinant *T. clavipes* MaSp1/MaSp2 proteins with collagen showed improved cell adhesion and proliferation of stem cells. Increased collagen‐contents enhanced differentiation of human decidua parietalis placental stem cells (hdpPSCs) into neuronal cells, whereas spidroin‐dominant fibers displayed better mechanical properties.^[^ ^137^ ^]^ An interesting biomimetic approach has been applied using fusion of the barnacle cement protein cp19k with MaSp1 from *Trichonephila clavata*. The fusion cp19k‐MaSp1 was then blended with poly L‐lactic‐co‐caprolactone (PLCL) and electrospun. The blended meshes exhibited enhanced adhesion capability of aluminum and glass surfaces, as well as improved tensile strength and ductility if compared to scaffolds just from PLCL.^[^ ^138^ ^]^

Spidroin‐based fiber meshes could be further functionalized upon incorporation of inorganic nanoparticles. Exemplarily, an electrospun nanocomposite made of spidroin with embedded cerium oxide nanoparticles has been created showing fluorescence and a decrease in electric resistance over time when subjected to cyclic loads depending on humidity. This nanocomposite has potential in sensing applications.^[^ ^139^ ^]^ Blending the recombinant MaSp and sodium hydrogen sulfide (NaHS) enabled nanofibrous membranes associated with high hemocompatibility and cytocompatibility capable of stably releasing H_2_S, playing an important physiological role in regulating the cytoprotective signaling processes.^[^ ^140^ ^]^

##### Coaxial Electrospinning for Bi‐ or Multifunctional Fiber Surfaces

Janus‐type nanofibers were achieved via coaxial electrospinning of two different dopes containing a cysteine‐tagged and an unmodified “inert” spidroin (Figure 5E). Binding gold nanoparticles (AuNPs) to such Janus fibers resulted in site‐selective immobilization of the AuNPs on the Cys‐tagged fiber part, due to high affinity between its thiol groups and the AuNP surface. The approach demonstrated the potential to realize complex bi‐ or even multifunctional structures with spatial resolution in the nanoscale for advanced energy applications.^[^ ^141^, ^142^ ^]^

The application of genetically modified spidroins and spidroin‐based blends is summarized in **Tables** 1 and **2**, respectively.

**Table 2:** Recombinant spidroins used in processing of electrospun blends.

*Origin*/recombinant silk protein	Blending component	Gained function/application	Refs.
*A. ventricosus* NTD from MaSp1, CTD from MiSp, RCD (W) from aciniform spidroin/NTW1–4CT	poly(L‐lactic‐co‐ε‐caprolactone) (PLCL)	improved mechanics improved biocompatibility with human umbilical vein endothelial cells (HUVEC), human epidermal growth factor (hEGF) delivery	[132, 134]
*A. ventricosus* NTD, CTD, RCD (R) and spacer (S) modules from MiSp/R1SR2 and NR1SR2C	poly(lactic‐co‐glycolic) Acid (PLGA)	enhanced proliferative and adhesive abilities of human bone mesenchymal stem cells (hBMSCs)/bone tissue engineering	[135]
*A. diadematus* ADF4/pNSR16 ± RGD	polycaprolactone (PCL)	improved cell adhesion of NIH‐3T3 cells	[129]
	polyvinyl alcohol (PVA)	in vivo tests in Sprague Dawley rats/wound dressings	[130]
	PCL, gelatin (Gt) and chitosan (CS) – (pNSR16/PCL/CS)/(pNSR16/PCL/Gt) bilayer	interaction with mesenchymal stem cells (MSC)/blood vessel tissue engineering	[271]
*A. diadematus* ADF4/pNSR32 ± RGD	PCL/gelatin	prolonged kinetic clotting time and reduced platelet adhesion/blood vessel tissue engineering	[131]
*N. clavipes* MaSp1 and 2/rS1/9 and rS2/12	PCL	directly reprogrammed neural precursor cell (drNPC) differentiation/nerve tissue engineering	[133]
*A. diadematus* MaSp2/eADF4(C16)	polylactide–polyglycolide (PLGA)	HaCaT (human keratinocytes) viability test, wound closure tests/wound healing applications	[136]
*T. clavipes* MaSp 1 and MaSp 2/(MaSp1)_24_, (MaSp2)_16_	collagen	viability, alignment and differentiation of human decidua parietalis placental stem cells (hdpPSCs)/nerve guide conduit scaffold	[137]
	cerium oxide nanoparticles	mechanically tunable electric resistance/sensors	[139]
*E. australis* MaSp1 (NTD and RCD (Rep), *A. ventricosus* MiSp CTD/NT‐2Rep‐CT	sodium hydrogen sulfide (NaHS) particles	H_2_S release – gas transmitter supporting wound healing.	[140]

### 2.3 Nanostructured Spidroin Films

#### 2.3.1 Spidroin Films as Materials’ Coatings

Coating made of spidroins can be processed out of organic solvents or aqueous buffers using drop casting, spin‐ or dip‐coating. Directly after drying, most spidroin coatings reveal prevalently random coil structures with minor α‐helical and β‐sheet components, hence reflecting the intrinsically unstructured state of the underlying spidroins in the soluble state. The solvent used and the drying conditions have severe impact on the secondary structure distribution in the coating. Hence perfluorinated solvents (HFIP and hexafluoroacetone) induce more α‐helices in comparison to aqueous buffers resulting in more random coil structures.^[^ ^143^, ^144^, ^145^, ^146^, ^147^ ^]^ Utilization of formic acid as a solvent, increased air humidity, and/or increased temperature during the drying process, in contrast, results in coatings that are enriched in β‐sheet structures.^[^ ^145^, ^146^, ^148^ ^]^ Consequently, to achieve spidroin coatings in a reproducible manner, strictly controlled processing conditions are required in terms of used solvents, temperature and humidity.^[^ ^149^ ^]^ Amorphous films (i.e., with low β‐sheet content) are water‐soluble hindering many applications. Hence, a transformation into β‐sheet‐rich structures has to be done using post‐treatment procedures including incubation in phosphate buffers,^[^ ^143^, ^144^ ^]^ alcohols,^[^ ^144^, ^146^, ^150^ ^]^ or heat treatments.^[^ ^148^, ^151^ ^]^ The conformational changes, which are often accompanied by an increased surface roughness,^[^ ^144^, ^145^, ^152^ ^]^ mainly occur in the poly‐Ala regions as determined using solid state NMR.^[^ ^147^, ^148^ ^]^ X‐ray diffraction indicated a wide distribution of β‐sheet crystallite sizes from 2 to 40 nm.^[^ ^145^ ^]^ Interestingly, it is possible to axially align the β‐sheets upon film stretching,^[^ ^147^ ^]^ and the β‐sheet rich spidroin films also possess enhanced stability against strong protein denaturing agents.^[^ ^144^ ^]^ Still, the spidroin films can be degraded by cell matrix proteases, and it's extend depends on the molecular weights of the spidroins.^[^ ^119^, ^153^, ^154^, ^155^ ^]^

Generally, the repeating units of spidroins are amphiphilic block‐copolymers comprising hydrophobic poly‐Ala motifs (block A) and hydrophilic Gly‐rich regions (block B),^[^ ^156^, ^157^ ^]^ especially in the case of MaSp‐ and MiSp‐based variants. Such block‐copolymers typically undergo phase separation in solvents or at interfaces, such as between solvent and air or solvent and substrate, depending on the block‐length ratio and Flory–Huggins interaction parameters, with similar blocks trying to maximize and dissimilar blocks trying to minimize their interactions.^[^ ^69^, ^158^, ^159^ ^]^ Unlike other proteins, the spidroins are typically composed only of ≈10 out of 20 naturally occurring amino acids including hydrophilic charged (Glu, Arg), uncharged (Gln, Ser), small (Gly, Ala) and large hydrophobic residues (Tyr). Phase separation, leading to the formation of nano‐ and microphases, have been reported for spidroins,^[^ ^69^, ^160^, ^161^, ^162^, ^163^, ^164^ ^]^ but also for silkworm fibroin,^[^ ^165^, ^166^ ^]^ as well as other protein‐based block copolymers.^[^ ^167^ ^]^

**Figure 6:**
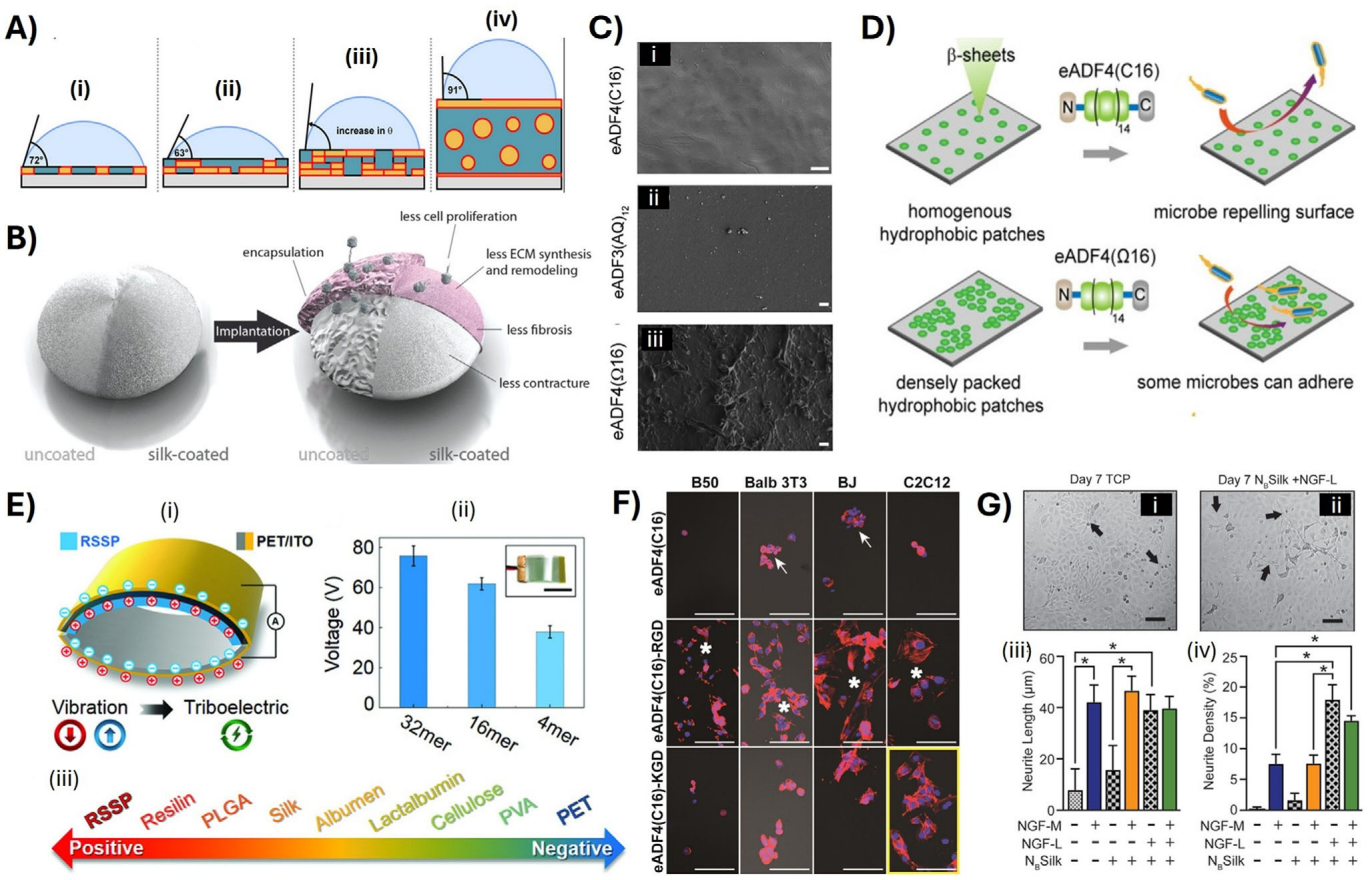
A) Structural assembly model of eADF4(C16) films dependent on the film thickness from the nano‐ to the microscale (simplified separation scheme), and the impact of the surface structures on wetting properties (demonstrated by a static water contact angle): (i) monolayered nanofilms showed 2D separation of the hydrophilic and hydrophobic spidroin sequences, (ii) bilayer nanofilms showed a poly‐Ala‐enriched phase at the underlying silicon substrate surface leading to exposition of the hydrophilic sequences at the protein/air interface, (iii) multilayered nanofilms yielded poly‐Ala‐rich surface/protein interfaces and β‐sheet rich protein/air interfaces which are hydrophobic, and (iv) microfilms showed microphase separation in the bulk film in which β‐sheet crystallites are separated at the protein/air interface as well as embedded as micelle‐like inclusions in the amorphous matrix. Reproduced with permission from Ref. [157]. B) Model of the bioshield function of a spidroin coating on silicone implants. The coating of medical grade silicone implants using eADF4(C16) resulted in a significant improvement of the implant's biocompatibility by effectively masking the implant's surface during the first months after implantation. Reproduced with permission from Ref. [153]. C) SEM images of films made of eADF3 and ADF4 variants after incubation with bacteria (exemplarily shown for *E. coli*). D) Model illustrating the mechanism of bacterial repellence of spidroin surfaces. Homogeneous hydrophobic patch distribution of eADF4(C16) and eADF3(AQ)12 yielded microbe repellence characteristics, whereas the absence of charge‐charge repulsions in the non‐charged eADF4(Ω16) variant led to dense packing of hydrophobic patches favoring the attachment of microbial cells. Reproduced with permission from Ref. [183]. E) Design of triboelectric nanogenerators (TENG) using spidroin films. (i) Schematic diagram of the TENG working principle illustrating the efficient ability of the spidroin films to lose electrons after contacting the polyethylene terephthalate (PET) layer. (ii) Outputs of TENGs based on spidroins differing in numbers of repetitive sequence motifs. (iii) Relative ability ranking from losing electrons (positive) to gaining electrons (negative) for various materials. Reproduced with permission from Ref. [189]. F) Cell adhesion (B50 neuronal cells (mouse), Balb 3T3 fibroblasts (mouse embryo), BJ fibroblasts (human skin), C2C12 myoblasts (mouse muscle)) on films made of different peptide‐tagged eADF4 variants, as indicated, demonstrating cytophobic, cytophilic and cell selective properties of the respective films depending on the functionalization of the spidroin. Cell nuclei and F‐actin cytoskeleton were stained using 4, 6‐diamidino‐2‐phenylindole (DAPI) (blue) and Phalloidin (red), respectively. Arrows point toward round cellular aggregates, and asterisks highlight clearly spread cells. The yellow box marks the enhanced selectivity of surfaces covered with the eADF4(C16)‐KGD variant toward C2C12 myoblast binding. Scale bars: 100 µm. Reproduced with permission from Ref. [193]. G) Evaluation of neurite outgrowth as a function of spidroin film modification. Representative micrographs (i and ii, scale bars: 100 µm) at indicated timepoints after the PC12 culturing on either tissue culture plastic (TCP) or on NBSilk pre‐loaded with NGF (NGF‐L). Optical images were analyzed to quantify neurite outgrowth and lengths depending on the indicated culturing conditions (iii). Reproduced from Ref. [195].

Upon spin‐coating of spidroins on substrates, the phase‐separation phenomena depend on the number of produced layers.^[^ ^157^ ^]^ β‐sheet structures mainly occurred at the substrate/protein interface, especially in very thin films. The β‐sheet content reached its maximum at a film thickness of ≈100 nm. In contrast, the surface properties (protein/air interface) of the spidroin films changed up to a thickness of 600 nm.^[^ ^157^ ^]^ When eADF4(C16) was cast into microfilms (thickness 1–2 µm), microphase separation occurred depending on the substrate's wettability (**Figure** **6**A). On hydrophilic surfaces, the hydrophilic blocks were oriented toward the substrate, and hydrophobic poly‐Ala patches were oriented toward the film/air interface. Vice versa, on hydrophobic substrates, the initial orientation of poly‐Ala crystallites led to redistribution of the phases, and subsequently more hydrophilic phases occurred on the spidroin film surface.^[^ ^157^, ^161^ ^]^ The presence of a nanocrystalline component associated with β‐sheet self‐assembly in thick films (>1 µm) enabled self‐healing of the spidroin coatings occurring through the rupture and reformation of the reversible hydrogen bonding between the β‐sheet nanocrystals at increased humidity.^[^ ^168^, ^169^ ^]^ The respective spidroins have been derived from MaSp sequences of the golden orb weaver *Nephila pilipes* and demonstrated extraordinary in situ self‐repair surpassing that of the spider *Cyrtophora moluccensis* and the silkworm *Bombyx mori*.

Coatings made of spidroins are promising because they are biocompatible, nontoxic and biodegradable, as well as they do not trigger an inflammatory response.^[^ ^170^ ^]^ Since most spidroins lack cell binding motifs,^[^ ^10^, ^171^ ^]^ the spidroin coatings can act as a protective, anti‐adhesive layer minimizing adverse effects of commercially available biomaterials.^[^ ^153^, ^172^, ^173^, ^174^ ^]^ eADF4(C16)‐coated implants and catheters display significantly reduced adhesion and proliferation of any cells as compared to non‐treated ones.^[^ ^153^, ^172^, ^175^ ^]^ One of the common problems of, e.g., silicone implants, is the formation of fibrous tissue when they are implanted in humans. Therefore, in a study reported by Zeplin et al.,^[^ ^153^, ^176^ ^]^ silicone‐based breast implants were coated with a micrometer thick film of eADF4(C16) using dip coating, drying and post treatment. After effectively masking the silicone surface, the coating significantly reduced capsule thickness, post‐operative inflammation, synthesis and remodeling of ECM, and expression of contracture‐mediating factors in vivo in comparison to uncoated ones (Figure 6B).

#### 2.3.2 Nanofilm Functionalization at Interfaces

Due to the amphiphilic character of spidroins^[^ ^177^ ^]^ nanostructured films can be obtained at liquid‐liquid and air‐liquid interfaces. Truncated recombinant spidroins derived from MaSp1 and MaSp2 (from *T. clavipes*) formed elastic nanofilms at the liquid–air interface.^[^ ^178^ ^]^ Hence, using the adjustment of hydrophobic (A‐block) and hydrophilic (B‐block) sequences in the spidroin variants, the formation of bowl‐shaped (1‐3 µm) or giant compound micelles (<50 µm) with porous membranes was observed in water/2‐propanol mixtures driven by the spidroin assembly on entrapped air bubbles, i.e., on liquid–air interfaces. The shape, the diameter, as well as the β‐sheet content increased with increasing numbers of poly‐Ala blocks (n = 1–6).^[^ ^179^ ^]^ eADF4(C16) has been studied at the water–oil interface using interfacial shear rheology, showing the high surface activity and fast formation of highly stable nanofilms.^[^ ^180^ ^]^ This behavior has been used to develop capsules at liquid‐liquid interfaces in water‐based emulsions, e.g., with toluene^[^ ^162^, ^181^ ^]^ or silicone oil.^[^ ^180^, ^182^ ^]^ Mechanically stable, micrometer‐sized capsules were produced in fast processes, being finished within 30 s. The size of these microcapsules (1–30 µm) could be controlled by adjusting the emulsification shear rates. The resulting capsule membranes allowed free diffusion of small molecules through the membrane, whereas larger macromolecules remained within the capsule. The capsules were found to have an average molecular‐weight cutoff of 27 kDa. The semi‐permeability of the capsules has been further used to develop an enzyme‐encapsuled protease‐protected system. Controllable activation of β‐galactosidase was achieved using α‐complementation, i.e., diffusion of a short peptide into the capsule containing an inactive dimer, structurally completing the enzyme and yielding the active tetrameric structure inside the capsules on demand.^[^ ^182^ ^]^

#### 2.3.3 Control of Coatings Using Spidroin Sequence Modifications

##### Spidroin Film Surface Activity Defined by Properties of the Underlying Spidroin Sequence

Controlling the spidroin sequence, i.e., the length of the A and B blocks and the protein charges influence the phase separation in spidroin coatings, specifically the distribution of β‐sheet crystallites within the amorphous matrix. A toolbox based on MaSp2 derivates of the European garden spider *A. diadematus* including eADF4(C16) and eADF3(AQ)_12_ as well as charged and uncharged variants^[^ ^62^, ^87^, ^155^ ^]^ allowed for studying the spidroin surfaces avoiding microbial interactions. A model for microbial interaction and repellence depending on the spidroin structure was developed. The size and distribution of surface‐exposed hydrophobic patches were engineered through intermolecular charge‐charge repulsion, as seen in the negatively charged eADF4(C16), or through the volume effect of the amorphous region in eADF3(AQ)12. Coatings made of such spidroins exhibited homogeneously distributed hydrophobic patches that were too small for microbial interactions, resulting in microbial repellence. However, the uncharged variant eADF4(Ω16) could not inhibit microbial interaction due to densely packed and less separated hydrophobic patches. Similarly, *B. mori* silk coatings, in contrast, with large hydrophobic patches supported microbial attachment and biofilm formation (Figure 6C,D). Importantly, this bacterial repellence could be transferred to different types of substrates and did not depend on surface microtopography,^[^ ^183^, ^184^, ^185^, ^186^ ^]^ as demonstrated recently on stainless steel and titanium implants using a *Galleria mellonella* *in vivo* model.^[^ ^187^ ^]^

Spidroin coatings have been further investigated in context of blood compatibility, biodegradability, serum protein adsorption, and cell adhesion.^[^ ^155^ ^]^ Among the tested spidroin derivatives, eADF4(C16) and eADF4(Ω16) revealed to be promising candidates for coatings of implants, medical devices, and long‐term storage depots of drugs or growth factors due to their biocompatibility, slow biodegradation, resistance to several proteolytic enzymes, low serum protein adsorption, microbial repellence, lack of blood coagulation, and cell repellence. Films made of eADF3(AQ)12 and eADF3(AQ)24 share these features but degrade faster, making them suitable for applications requiring quick biodegradation, such as drug or antibiotics delivery. Positively charged eADF4(κ16) coatings promoted serum protein adsorption, blood coagulation, and cell adhesion, making them ideal for wound dressings.^[^ ^93^, ^155^, ^183^, ^188^ ^]^

Apart of the biomedical applications, spidroin‐based films showed suitable properties for triboelectric nanogenerators (TENGs) with conductive synthetic polymer‐based substrates, such as poly(ethylene terephthalate) (PET) doped indium tin oxide (ITO) (Figure 6E). Whereas the conductive polymers are typically highly electronegative materials, spidroin‐based materials are usually prone to losing electrons, inducing the triboelectric effect. The possibility to control the molecular weight of spidroins was decisive in the improvement of the TENG properties. Such coatings showed significantly increased potential differences on PET‐substrates significantly enhancing the power density in comparison to other protein‐based materials, such as fibroin, keratin, collagen, or gelatin.^[^ ^189^, ^190^, ^191^ ^]^

##### Spidroin Fusions with Peptide Tags for Bio‐Selective Adhesion

As mentioned above, spidroins have been fused with an RGD‐peptide inducing integrin‐mediated cell adhesion.^[^ ^125^, ^126^, ^192^ ^]^ Systemic studies testing eADF4‐based variants with diverse cell‐binding peptides regarding primary cell adhesion of various cell types^[^ ^192^, ^193^ ^]^ showed that functionalization with a KGD‐peptide allowed selective attachment and growth of myoblasts, which could also be confirmed using co‐culture experiments with neuronal cells (Figure 6F).^[^ ^193^ ^]^ Functionalization with an REDV‐peptide exhibited endothelial cell selectivity.^[^ ^194^ ^]^ IKVAV‐ fusion with 4RepCT (consisting of four repeat sequences (4Rep) followed by a C‐terminal domain (CT) from *Euprosthenops australis* MaSp1) allowed to process films specifically supporting adhesion of Schwann cells,^[^ ^192^ ^]^ whereas vitronectin‐derived peptide fusions supported adhesion and neurite growth of dorsal root ganglion cells.^[^ ^127^ ^]^ Apart from MaSp constructs, aciniform silk variants (NBSilk) were terminally furnished with heptapeptide motifs binding the neurotrophic factor and nerve growth factor‐β (NGF). The films made thereof showed sequestering capability of the factors enhancing differentiation, neurite density and outgrowth in neuronal cells (Figure 6G).^[^ ^195^ ^]^

Films of spidroins fused with specific antimicrobial tags, such as cationic antimicrobial peptides including human neutrophil defensins (HNP)^[^ ^150^, ^196^ ^]^ or hepcidin,^[^ ^197^ ^]^ and heparin binding peptides have been prepared to actively combat bacterial infestation on the respective spider silk coatings.^[^ ^198^ ^]^ Considering the interaction with inorganic particles, specific affinity peptides, e.g. from bone extracellular matrix proteins, such as bone sialoprotein^[^ ^199^ ^]^ or osteopontin,^[^ ^200^ ^]^ have been fused to spidroins targeting the binding of silver,^[^ ^201^ ^]^ gold, titanium dioxide,^[^ ^202^ ^]^ silica,^[^ ^203^, ^204^ ^]^ or hydroxyapatite nanoparticles.^[^ ^205^ ^]^

An overview on the applicability of functionalized spidroin‐based coatings is provided in Table 1.

### 2.4 Nanostructured Surfaces via Self‐Assembly of Fibrils

#### 2.4.1 Solution versus Surface‐Based Self‐Assembly of Spidroins

**Figure 7:**
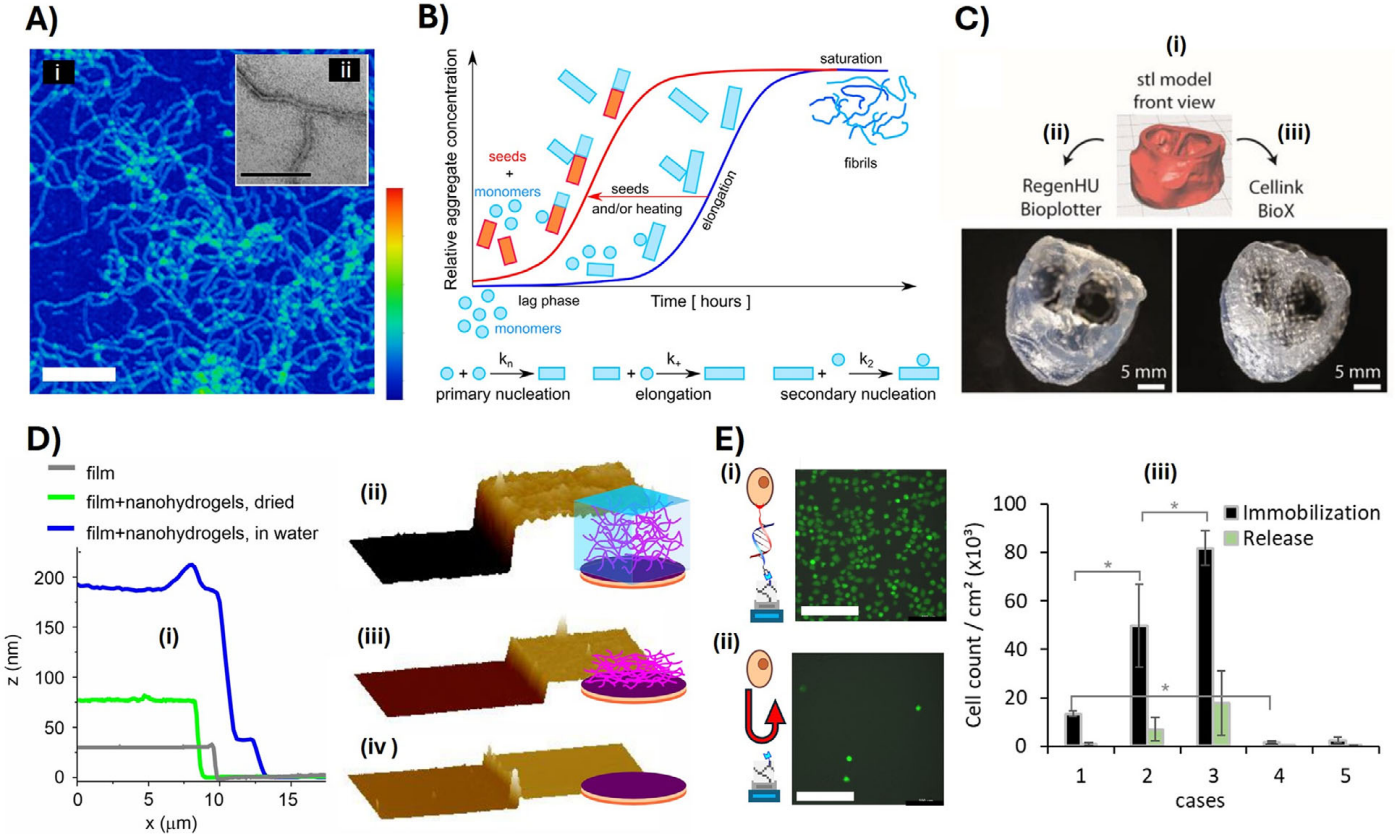
A) The morphology of eADF4(C16) fibrils assembled from 15 µm eADF4(C16) in 150 mm KPi buffer (pH 8) was visualized using atomic force microscopy (AFM, height image) Vol.: 57, pages: 831–843, (i)) and transmission electron microscopy (TEM, (ii)). The AFM image (i) shows characteristic fibril entanglements, while the TEM image (ii) reveals fibril branching. The color scale in (i) indicates heights from 0 nm (deep blue) to 22 nm (deep red). Scale bar: 500 nm in (i), 100 nm in (ii). B) The schematic overview illustrates the basic mechanisms of the assembly of eADF4(C16) into fibrils. Experimental data – measured as changes in turbidity over time – were analyzed using AmyloFit^[^ ^276^ ^]^ at various protein concentrations (10–50 µm) and temperatures (20–40 °C). The results revealed that, in addition to primary nucleation, secondary nucleation significantly contributed to fibrillation kinetics. During the initial lag phase, monomers oligomerize to form primary nuclei, which then enter the elongation phase characterized by rapid, exponential fibril growth through monomer addition. In secondary nucleation, monomers interact with the surface of existing fibrils, accelerating the fibril formation. Both the addition of seeds (shortened fibrils by ultrasound) and elevated temperatures further enhanced the rate of fibril assembly. Reproduced with permission from Ref. [210]. C) Shape fidelity of dispense plotted spider silk hydrogels was evaluated using a 3D model of a human aortic valve (STL file source: Cellink, see (i)). The structure was printed using 3% eADF4(C16) hydrogels on two different bioprinters: RegenHU's 3D Discovery and Cellink's BioX ((ii) and (iii), respectively). The most notable difference was the closed layers produced by the Discovery printer compared to the open pores observed using the BioX printer. These variations were attributed to differences in the G‐codes generated for the same 3D structure. Reproduced with permission from Ref. [219]. D) The swelling behavior of nanohydrogels assembled on a spin‐coated nanofilm – both composed of eADF4(C16) – was analyzed. Scratches introduced in the assembled layers (ii–iv) were examined using AFM. Cross‐sectional profiles (i) revealed the thickness of the nanofilms (≈30 nm, gray line) and the nanohydrogel in both dry (≈75 nm, green line) and swollen (≈200 nm, blue line) states. Reproduced with permission from Ref. [226]. E) The antifouling performance of DNA‐functionalized nanohydrogels was demonstrated using DNA‐assisted cell immobilization and release. Fluorescence microscopy after live‐staining revealed that complementary DNA‐labeled cells (i) exhibited high surface densities, whereas unmodified cells (ii) showed minimal surface interactions. The cell densities were quantified from fluorescence images following immobilization (black bars) and release (green bars) for cells labeled with 0.5, 2, and 5 µm DNA (cases 1–3) on DNA‐modified nanohydrogels and compared to DNA‐modified (case 4) and native cells (case 5) on unmodified nanohydrogels. In cases 1–3, release was triggered by introducing a competitive DNA probe. Scale bars: 100 µm. Reproduced with permission from Ref. [233].

Spidroin self‐assembly into nanofibrils is sequence specific. Nanofibrils could be produced from short spidroin inspired peptides, such as GAGAAAAAGAGA, as well as from the spidroin pS(4+1) derived from *T. clavipes* MaSp, which both spontaneously formed β‐sheet‐rich fibrils in aqueous buffers within several hours.^[^ ^206^, ^207^ ^]^ eADF4(C16) represents again the best studied spidroin in this context (**Figure** **7**A). The fibrils, which formation is typically induced at low protein concentration (1 mg mL^−1^) by phosphate ions (< 300 mm), showed a high content of β‐sheets (40%) and a cross‐β conformation.^[^ ^23^, ^208^ ^]^ Besides phosphate, further kosmotropic anions such as sulfate and fluoride could initiate eADF4(C16) fibril formation, whereas the rate constant of fibril growth correlated with the position of the ions in the Hofmeister series.^[^ ^82^, ^209^ ^]^ Detailed studies revealed a nucleation mechanism with slow oligomerization and nucleus formation and a fast growth phase. Secondary nucleation, i.e., induction of nuclei formation on fibril surfaces, appeared as further factor in the assembly kinetics (Figure 7B).^[^ ^82^, ^210^ ^]^ The self‐assembly in solution is very robust, tolerating diverse protein modifications, such as variation in number of repeats in case of eADF4(Cx) (x = 2, 4, 8, and 16 (see also Figure 3 for molecular design),^[^ ^23^ ^]^ chemical conjugation with nucleic acids,^[^ ^211^ ^]^ or genetical fusion with globular proteins, such as esterase or GFP.^[^ ^212^ ^]^ The self‐assembly mechanism is, however, highly sequence specific, i.e., no cross seeding with other fibrous proteins^[^ ^82^, ^83^ ^]^ or even with the sequentially related ADF4‐charge variants has been observed. Importantly, the fibril formation is not related to the above‐described formation of nanofibrillar structures in the fibers. The nanofibril assembly from highly concentrated spinning dopes takes place within milliseconds, and the fibrils as well as the β‐sheet structures are aligned with the fiber axis. In contrast, the self‐assembly from diluted solutions results in nanofibrils in a nucleation‐based process in hours or days, and the β‐sheets are perpendicularly oriented to the fibril axis.

#### 2.4.2 Spidroin Hydrogels and Bioprinting

Based on nanofibrils, spidroins can further assemble into hydrogels at protein concentrations above 1% w/v.^[^ ^213^, ^214^ ^]^ eADF4(C16)‐based hydrogels represent a hierarchically ordered, supramolecular polymer network with visco‐elastic properties and adjustable maximal shear stress between 200 and 2000 Pa, depending on the spidroin concentration (3–7% w/v).^[^ ^213^, ^215^ ^]^ Importantly, the physical crosslinking also allowed a shear thinning behavior which made the spidroin hydrogels bio‐printable without further additives (Figure 7C).^[^ ^216^, ^217^, ^218^, ^219^ ^]^ Hence, this feature enabled fine grid structures to be deposited on a surface, which cure immediately so that multilayer scaffolds could be produced. In addition to the mechanical stability during 3D bioprinting, biological activity/cell‐specific interactions (associated with slow biodegradation) are important. For example, murine fibroblasts and human epithelial pancreatic cell lines (BxPC‐3) or human embryonic kidney cells (HEK293) were encapsulated and co‐printed in spidroin hydrogels, with cell survival rates of over 90% during the 3D bioprinting process.^[^ ^216^, ^219^ ^]^ The hydrogels enabled also efficient encapsulation of HEK293 producer cells stably secreting the highly traceable biological reporter TNFR2‐Fc‐GpL, a fusion protein consisting of the extracellular domain of TNFR2, the Fc domain of human IgG1, and luciferase as a reporter domain. eADF4(C16) and eADF4(C16)‐RGD hydrogels promoted HEK293 cell growth and allowed stable fusion‐protein production over 14 days.^[^ ^220^ ^]^ To generate gradient biomaterial constructs, a numerical in silico approach was used to simulate gradient flow formation. After the optimization of the process using a computational setup, spidroin hydrogels were gradually printed with fluorapatite and encapsulated fibroblasts generating gradient materials suitable for, e.g. tendon/bone interface engineering.^[^ ^221^ ^]^ In a recent study, hydrogels made of interpenetrating networks of collagen‐I and eADF4(C16)‐RGD nanofibrils exhibited synergistic and tunable mechanical properties. Composite hydrogels allowed for cell adhesion and spreading of fibroblasts, C2C12 myoblasts, and human induced pluripotent stem cell (hiPSC)‐derived cardiomyocytes, and they were resistant to shrinkage mediated by the cells. Myoblast differentiation and fused myotube formation were observed in such composite hydrogels, and hiPSC‐cardiomyocytes could be long‐term cultured showing contractibility for up to 95 days in cell culture.^[^ ^222^ ^]^

Vascularization of silk‐based scaffolds is a crucial step for the generation of bioartificial tissues and, thus, for clinical applications. The above‐mentioned AV‐loop model (Chapter 2.2) was also used to implant hydrogels made of unmodified and RGD‐modified eADF4(C16). Biocompatibility and efficient vascularization were confirmed, which was even better than in other hydrogels such as those made from fibrin.^[^ ^223^, ^224^ ^]^

#### 2.4.3 Self‐Assembled Nanofibrillar Coatings

The formation of eADF4‐based nanofibrils on surfaces via phosphate‐triggered self‐assembly has been explored either using surface‐induced nucleation on immobilized spider silk layers (Figure 7D),^[^ ^82^, ^225^, ^226^ ^]^ or using the spidroin's is amphiphilic properties on glass, stainless steel, silicon, and TiO_2_ substrates.^[^ ^227^ ^]^ Nanofibrillar coatings made of 4RepCT showed swelling behavior, adjustable thickness and viscoelastic properties.^[^ ^228^, ^229^, ^230^, ^231^ ^]^ The interfacial water‐air behavior of this protein allowed for the development of free‐standing, protein‐permeable nanomembranes with an internal nanofibrillar structure supporting an engineering strain of over 220%.^[^ ^232^ ^]^

The swelling behavior of the nanofibrillar coatings resulted in low adhesion forces, which could be used in repelling cells from surfaces. The resistance of eADF4(C16)‐based nanofibrillar coatings to bacterial fouling was investigated using *Staphylococcus epidermidis* and *E. coli* showing reduced bacterial adhesion, hence being promising for improving the performance of indwelling implants and devices.^[^ ^227^ ^]^ Such nanohydrogel coatings have also been tested with normal and cancer cells, showing very low binding affinities (Figure 7E) as well,^[^ ^233^, ^234^ ^]^ which enabled development of cell‐selective binding modalities upon aptameric functionalization of the coatings, as described in the next section.

#### 2.4.4 Functionalization of Fibrillar Coatings Made of Spidroins

An RGD‐modified 4RepCT variant (FN‐4RepCT) was used to form nanofibrillar coatings on titanium, stainless steel, and hydroxyapatite substrates to support adhesion of human primary cells, implying the potential to improve implant performance and acceptance by the body.^[^ ^228^ ^]^ Further, 4RepCT was fused with six cationic lysins (pLys‐4RepCT) or with the human galectin‐3 carbohydrate recognition domain (hGal3‐4RepCT) to gain mucoadhesive properties.^[^ ^235^ ^]^ A two‐step functionalization approach was used, first to assemble coatings from sortase‐tagged (poly‐Gly) spidroin, which was second conjugated with enzymes, such as the biofilm‐dispersal dispersin B (DspB) and peptidoglycan degrading endolysins PlySs2 and SAL‐1, using sortase A‐mediated coupling. Such coatings reduced the biofouling properties of *Staphylococcus aureus*.^[^ ^236^ ^]^ Using a fusion with an antibody‐binding Z domain (Z‐4RepCT) was demonstrated to capture the antibodies on a variety of materials while simultaneously reducing nonspecific adsorption.^[^ ^237^, ^238^, ^239^ ^]^

eADF4(C16)‐based nanohydrogel coatings were cell adhesive when using the RGD‐modified variant.^[^ ^233^, ^234^ ^]^ Moreover, ADF4(C16) could be chemically conjugated with short oligonucleotides, using coupling pairs with an azido and an alkyne linker, respectively. The nucleic acid moieties did not hinder the protein self‐assembly into fibrils, whereas the conjugated nucleic acid strands were exposed on the fibril surface,^[^ ^211^, ^226^ ^]^ allowing functionalization of the nanofibrillar coatings with nucleic acids called aptamers possessing the capability to bind specific targets, such as thrombin. Such coating allowed for the formation of enzymatic depots which could be released on‐demand by an aptamer structural switch.^[^ ^226^ ^]^ An aptameric modification targeting cancer cell markers, i.e., overexpressed membrane proteins, led to cell‐specific immobilization of non‐adherent leukemia T cells (Jurkat), as well as adherent cervix carcinoma (HeLa) and neuroblastoma (Kelly) cells.^[^ ^233^ ^]^

Spidroins and their modifications used in preparation of self‐assembled nanofibrillar hydrogels and coatings are summarized in Table 1.

### 2.5 Patterning of Film and Nanofibrillar Spidroin Surfaces

The formation of films or self‐assembled nanofibrillar coatings, as described above, was combined with photo‐ or soft‐lithography techniques to create nano‐ and micro‐patterned surfaces. The micro and nanofabrication technologies are integral to the development of miniaturized systems and devices.^[^ ^240^ ^]^ The main limitations in using integrated circuit‐oriented nanofabrication technologies with biological systems, however, lie in harmonizing these technologies to alleviate the inherent mismatch between biological (soft–wet) and nonbiological (hard–dry) components. Moreover, it has been recognized that biomedical applications require precise reconstructions of the complex biological microenvironment, mimicking 3D geometries of biological cues at nanometer and micrometer scales.^[^ ^241^, ^242^ ^]^ Silk proteins originating from silkworms and spiders provide “green” alternatives to synthetic materials with advantages such as good mechanical properties (being detrimental to creating sophisticated 3D nanostructures without collapsing), high biocompatibility, ease of functionalization, controllable water‐solubility, and tunable degradation rates, all of which offer numerous opportunities for processing, biological adaptation, and integration of lithographic techniques as exemplified using fibroin surfaces employed in nanoimprinting lithography,^[^ ^243^ ^]^ soft lithography,^[^ ^244^ ^]^ dip‐pen nanolithography,^[^ ^245^ ^]^ laser machining^[^ ^246^ ^]^ or forming radiation‐sensitive materials via electron beam lithography (EBL)^[^ ^247^ ^]^ or photolithography.^[^ ^248^, ^249^ ^]^

#### 2.5.1 Spidroin Coatings as Green Resists

**Figure 8:**
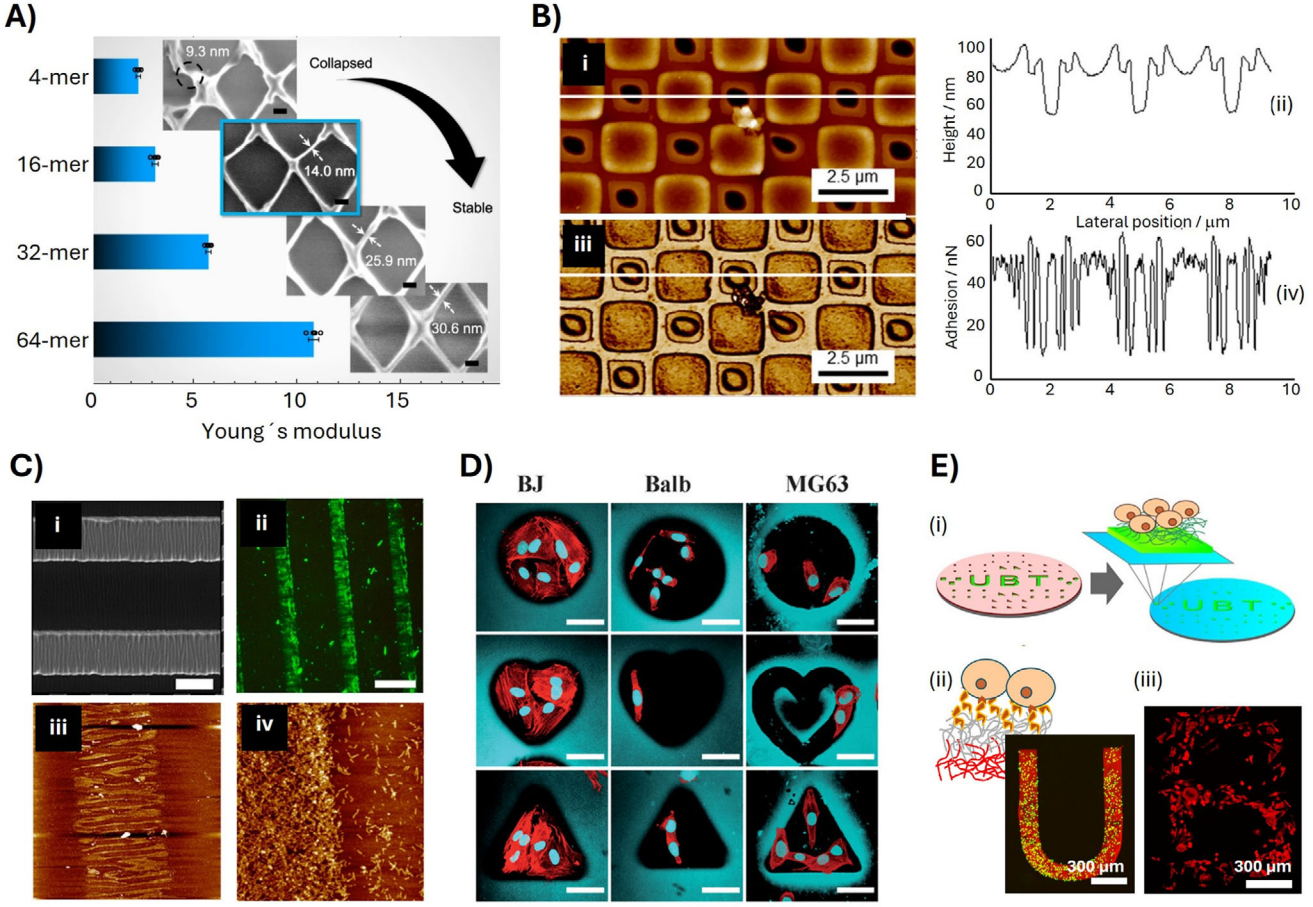
A) Mechanical properties and the minimum feature sizes achieved using EBL on films made of different spidroins differing in numbers of repeating sequences (4‐mer – 64‐mer). The fidelity of the structure increased with the spidroin length and correlated with the Young's modulus. Scale bars in SEM images 100 nm. Reproduced with permission from Ref. [250]. B) A polystyrene (PS) mask was deposited using capillary transfer lithography (CTL) on eADF4(C16) films. Then patterned regions were formed on the spidroin film using solvent (MeOH)‐induced conformational transition of the non‐masked regions. (i) Topography scan and (iii) adhesion map are presented with corresponding cross sections of the heights (ii) and adhesion forces (iv). Reproduced with permission from Ref. [152]. C) Confocal laser scanning microscope images of cell adhesion (Balb 3T3 fibroblasts (mouse embryo), BJ fibroblasts (human skin, MG63 fibroblasts (human osteosarcoma)) within differently shaped micro‐indentations in patterned eADF4(C16) films showed different accommodation of the cells depending on cell type and the shape of the indentation. Cell nuclei (blue) and F‐actin cytoskeleton (red) were visualized using DAPI and phalloidin‐red staining, respectively (scale bar: 50 µm). Reproduced with permission from Ref. [261]. D) Microcontact patterning of spidroin nanofibrils: (i) Scanning electron microscopy of an irregularly wrinkled PDMS stamp used to pattern a link^DNA^‐eADF4(C16) conjugate on a dextran/cap^DNA^ modified substrate (scale bar: 20 µm). (ii) Fluorescence microscopy patterns after locally induced fibrillization of fluorescein‐labeled eADF4(C16) on a pre‐stamped surface (scale bar: 200 µm). (iii) and (iv) AFM scans of the patterns in (ii) at different length scales (AFM scan widths are 50 and 3 µm in iii and iv, respectively). Reproduced with permission from Ref. [225]. E) DNA‐assisted cell immobilization on nanofibrillar micropatterns. (i) Schematic representation of the micropatterning approach using a positive‐tone photoresist (reddish) enabling the creation of microwells for spatially defined immobilization and self‐assembly of the DNA‐modified spidroins (green) which could be exposed to cells after striping the photoresist on a PEG‐blocked surface (pale blue). (ii) HeLa cells immobilized on double‐labeled micropatterns. Nanohydrogels of TAMRA‐eADF4(C16) (red) were assembled first in microwells, followed by assembly of azido‐modified ADF4(C16) enabling conjugation with DBCO‐aptamers. The approach resulted in aptamer‐functionalized U‐patterns being tested in cancer‐specific cell targeting, as demonstrated with HeLa cells. (iii) HeLa cells showed spreading on a B‐shaped micropattern after 24 h of incubation. The cells were stained with Calcein‐AM for viability in green in (ii) and for actin cytoskeleton with phalloidin‐red in (iii) and visualized using fluorescence microscopy. Reproduced with permission from Refs. [233, 265].

Spidroin coatings have been developed as green resists enabling precise nanostructuring using electron and ion beam lithography (EBL and IBL).^[^ ^250^, ^251^ ^]^ The process is entirely water‐based, starting with the formation of a spidroin film from aqueous solutions and ending with simple development of the exposed patterns in water. Controlling the beam energy on the spot, the structural spidroin transitions from an amorphous state (soluble in water) to a β‐sheet rich state (insoluble in water) could be achieved. Using coatings made of MaSp1 variants from *T. clavipes* and accurately directed ion and electron beam interactions with the protein's matrix, enabled the creation of well‐defined 2D and 3D nanopatterns. Control over spidroin sequence and molecular weight (**Figure** **8**A) provided lithographic resolutions approaching the molecular limits and surpassing that of polymethyl methacrylate (PMMA). The spidroins could be further functionalized with a variety of chemical and biological dopants such as fluorescent dyes, enzymes, and antibodies through simple mixing before their processing into nanostructured coatings. EBL and IBL in combination enabled the generation of nanoscaled structures with minimum feature sizes between 13 and 50 nm. Importantly, spidroins ‐ as the resist ‐ offered good mechanical properties being essential to build sophisticated nanoarchitectures with high aspect ratios and structural stabilities.^[^ ^250^, ^251^ ^]^

#### 2.5.2 Soft Lithography Techniques Applied on Spidroin Coatings

To overcome limitations of light, ion, and electron beam lithography, such as nanopatterning on large areas (i.e., > 1 cm^2^), curved surfaces, or surfaces made of irradiation‐insensitive materials, conceptually simple lithography methods including embossing, molding, or stamping have been developed to serve as the basis for nanofabrication of proteins.^[^ ^252^ ^]^ Based thereon, high resolution nanostructures,^[^ ^253^ ^]^ microfluidic devices^[^ ^254^ ^]^ and patterning of protein‐based materials can be achieved.^[^ ^255^, ^256^, ^257^, ^258^ ^]^

The principles of capillary transfer lithography (CTL) and solvent‐assisted microcontact molding (SAMIM) on polystyrene (PS) have been utilized in combination with eADF4(C16) nanocoatings to create patterns on spidroin films (Figure 8B). By employing CTL or SAMIM, PS nanopatterns have been developed in the upper layer, thereby preventing the exposure of the spidroin to methanol. This induced high β‐sheet content in the unmasked areas and revealed fine sub‐micrometer details of morphological and mechanical changes in the patterned regions after PS stripping.^[^ ^152^ ^]^

Poly(dimethylsiloxane) (PDMS) stamps, developed originally for microcontact printing, were also used for micromolding in capillaries (MIMIC), allowing for the formation of complex microstructures.^[^ ^259^ ^]^ Using MIMIC in combination with coatings based on spidroin and lacewing egg stalk proteins, patterned scaffolds have been made to orient cells, such as mouse fibroblasts and myoblasts, on surfaces.^[^ ^260^ ^]^ Microstructures made of eADF4(C16) using replica molding (REM) enabled selective cell adhesion on the usually non‐cell‐adhesive material (see above). The surface microindentations showed different shapes and dimensions, including grooves, circles, squares, and stars, which served as anchoring points, supported cell alignment, and enabled contact guidance (Figure 8C).^[^ ^261^ ^]^ Conversely, REM‐micropatterned eADF4‐based coatings substantially restricted the attachment, growth, and microbial colonization of pathogenic bacteria as well as fungi.^[^ ^183^ ^]^ Combining soft‐lithography techniques with spidroin self‐assembly allowed for the formation of patterned arrays comprising nanofibrillar structures (Figure 8D). eADF4(C16) was conjugated with short DNA,^[^ ^211^ ^]^ and the resulting conjugates were hybridized on silica surfaces functionalized with complementary DNA with a spatial precision defined by microcontact printing. The immobilized spidroin triggered surface‐localized nucleation and growth of fibrils, which finally copied the pattern of the sub‐microstructured wrinkled PDMS stamp.^[^ ^225^ ^]^ Specific propensity of 4RepCT to form oriented conformations on hydrophobic surfaces^[^ ^231^ ^]^ allowed for the generation of patterned coatings, nanowires or sheets using superhydrophobic micropillars and controlled spidroin drying.^[^ ^262^, ^263^ ^]^

#### 2.5.3 Patterning of Spidroin Nanostructures Using Photolithography

Employing positive‐tone photolithography with a commercial photoresist, arbitrarily shaped micro‐wells have been created with chemically active bottoms suited for the chemical immobilization of eADF4(C16) and application of the principle of surface‐triggered nucleation. Prolonged fibrillization in the microwells led to the formation of nanoscaled networks which copied the shape of the microwell with high fidelity after the photoresist stripping (Figure 8E(i)). The robustness of the network allowed for repetitive photolithography/self‐assembly cycles resulting, in the case of a fluorescently labeled spidroin, in adjustably positioned functionalized microstructures.^[^ ^264^ ^]^ Application of the approach on nucleic acid‐modified spidroin‐based nanohydrogels enabled the creation of cell‐microarrays employing binding modalities between DNA‐modified surfaces and DNA‐modified cells and specific aptamer‐cancer cell markers, respectively (Figure 8E(ii,iii)).^[^ ^233^, ^234^ ^]^

## 3 Summary and Outlook

The intrinsic properties of spidroin‐based materials, such as biocompatibility and mechanical robustness, along with the ability to use both genetic modifications and chemical conjugations, even in parallel if required, allows for the creation of customized spidroins and nanostructured surfaces made thereof with pre‐defined properties opening paths toward many biomedical and technical applications. Accessibility of water‐based processing of spidroins provides a sustainable alternative to processing of synthetic materials. Further, spidroin‐based nanostructured surfaces are ideal for including environmentally sensitive biochemical components such as enzymes, growth factors, antibodies, or aptamers. Such nanostructured and functionalized spidroin‐hybrid surfaces will be suitable for applications in tissue engineering, wound healing, and drug delivery systems. The combination of spidroins with manufacturing techniques like electrospinning and lithography additionally opens possibilities for creating new complex nanostructured hierarchically organized surfaces and coatings, which could be used in areas, such as bioelectronics, battery applications, catalysis, filtration of bioactive components and protective antimicrobial coatings of biomedical devices or implants.

Despite these promising developments, several challenges remain: 1) The scalability and consistency of recombinant spidroin production is still not solved in many cases, which currently depend heavily on the specific spidroin sequence. 2) The structure‐function relationships in spidroins are complex and not yet fully understood. 3) Controlling self‐assembly dynamics remains difficult due to the high sensitivity of spidroins to environmental factors such as pH, temperature, ionic strength, and shear forces. 4) Functionalization strategies often introduce trade‐offs between self‐assembly and surface activity; for example, adding peptide tags for cell binding or nanoparticle capture, or modifying repeat content, can impact β‐sheet formation and hence the stability of the coating and/or surface exposure of the tag.

Achieving sub‐micron resolution in patterning is also technically challenging. A shift is needed from traditional lithographic techniques to approaches that are compatible with protein sensitivity and can be operated under mild, aqueous, and physiological conditions. Promising strategies could include enzyme‐mediated patterning and VIS‐light‐activated chemistries that do not impair protein chemistry. Furthermore, long‐term bio‐integration and the stability of self‐assembled nanostructures under physiological conditions remain areas of active investigation. Finally, standardized protocols for functional testing in realistic environments are still lacking.

To address these challenges, future research should focus on several strategic directions: developing synthetic biology tools to improve recombinant spidroin yield and purity; applying multiscale modeling and machine learning to better understand sequence‐assembly relationships; and designing stimuli‐responsive spidroins for dynamic functionality and tunable degradation. Enhancing compatibility with high‐resolution lithographic and additive manufacturing methods, investigating long‐term biostability and immune responses, and promoting interdisciplinary collaboration will be crucial for advancing the field.

In summary, spidroin‐based materials represent a cutting‐edge frontier in bioinspired nanotechnology. With sustained research and cross‐disciplinary innovation, they hold the potential to revolutionize the development of sustainable, functional surfaces for next‐generation biomedical and technological applications.

## Conflict of Interest

TS is co‐founder and shareholder of AMSilk GmbH.
